# Synthesis and biological evaluation of truncated derivatives of abyssomicin C as antibacterial agents

**DOI:** 10.3762/bjoc.15.147

**Published:** 2019-07-02

**Authors:** Leticia Monjas, Peter Fodran, Johanna Kollback, Carlo Cassani, Thomas Olsson, Maja Genheden, D G Joakim Larsson, Carl-Johan Wallentin

**Affiliations:** 1 Department of Chemistry and Molecular Biology, University of Gothenburg, Kemigården 4, 412 96, Gothenburg, Sweden; 2 Centre for Antibiotic Resistance Research (CARe), University of Gothenburg, Gothenburg, Sweden; 3 current affiliation: Hit Discovery, Discovery Sciences, R&D BioPharmaceuticals, AstraZeneca, Gothenburg, Sweden; 4 Department of Infectious Diseases, Institute of Biomedicine, The Sahlgrenska Academy at the University of Gothenburg, Guldhedsgatan 10, 413 46, Gothenburg, Sweden

**Keywords:** abyssomicin C, antibiotic, antibiotic resistance, MRSA, truncated natural products

## Abstract

The synthesis and antibacterial activity of two new highly truncated derivatives of the natural product abyssomicin C are reported. This work outlines the limits of structural truncation of the natural product and consequently provides insights for further structure–activity relationship studies towards novel antibiotics targeting 4-amino-4-deoxychorismate (ADC) synthase. Specifically, it is demonstrated that the synthetically challenging bicyclic motif is essential for activity towards methicillin-resistant *Staphylococcus aureus* (MRSA).

## Introduction

**Figure 1:**
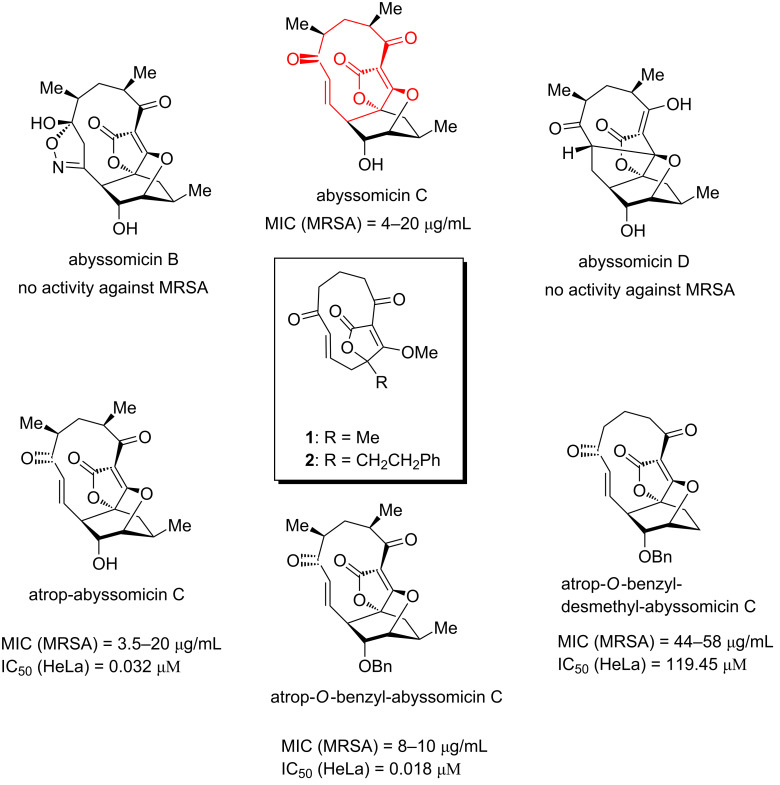
Structure of abyssomicins C, B, D, atrop-abyssomicin C, atrop-*O*-benzyl-abyssomicin C and atrop-*O*-benzyl-desmethyl-abyssomicin C with their biological activities, and the truncated derivatives **1** and **2**. In red: part of abyssomicin C that is maintained in the truncated derivatives [4,7–10].

Antibiotic resistance is a major threat to global health, and numerous actions are presently taken in both academia and industry to combat this major societal challenge. There are many different approaches to develop new antibiotics, but the reevaluation of known natural products (NP) and the identification of new NPs and derivatives thereof is considered an especially important pathway in the search for more robust antibiotics [1–2]. The main reasons are the inherent high selectivity and appropriate physicochemical properties that NPs typically show [3]. Abyssomicin C (AbC) is an NP with antibacterial activity that was isolated from the marine actinomycete strain *Verrucosispora* AB-18-032 in 2004 [4–5]. It shows antibacterial activity against Gram-positive bacteria, including resistant pathogens such as methicillin- and vancomycin-resistant *Staphylococcus aureus* (MRSA and VRSA) with minimum inhibitory concentration (MIC) values of 4 and 13 µg/mL, respectively [4]. AbC belongs to a small family of NPs and two congeners of this family lacking the enone functionality, abyssomicins B and D, were discovered at the same time, and these compounds did not show any antibacterial activity (Figure 1). Consequently, the enone moiety of AbC was proposed to play an essential role in the antibiotic activity. Indeed, in a subsequent study it was shown that AbC has a unique mechanism of action amongst NPs: it is a covalent inhibitor of 4-amino-4-deoxychorismate (ADC) synthase, which is the enzyme that catalyzes the conversion of chorismate and glutamine into ADC and glutamate, the first step in the biosynthesis of *p*-aminobenzoic acid (PABA) in bacteria [6]. Specifically, AbC binds via a Michael addition between a cysteine residue in the immediate proximity to the active site and the enone moiety of AbC. The PABA pathway is essential in bacteria but absent in humans, making AbC a promising compound for further development towards an antibiotic drug candidate.

Because of its intriguing structure and antibacterial activity, several synthetic studies [11–14], formal [15] and total [7–9,16–17] syntheses of AbC and its slightly more active atropisomer (atrop-AbC) have been reported in the last ten years. Due to its complex structure, these total syntheses are long and thus not compatible with further drug development studies.

An advantageous approach to strategically truncate complex molecules and thereby simplify their synthesis is referred to as function-oriented synthesis (FOS) [18]. This strategy allows for a more rapid drug development process, and it has previously been applied to AbC. Accordingly, structure–activity relationship (SAR) studies have been performed, which indicate that benzylation of the hydroxy group and the complete demethylation of the core structure of AbC retain the antibiotic activity towards MRSA while simultaneously decrease cytotoxicity towards various human cell lines [9–10].

Based on these observations, and in line with the principles of FOS, we hypothesized that further simplification could be compatible with retained activity and consequently also a more rapid and exhaustive SAR exploration. Specifically, we identified truncated derivative **1** as the most simplified structure that still includes all structural features that have been previously found to be essential for activity. This truncated derivative still has the tetronate functionality and the enone-equipped 11-membered ring system but lacks the oxabicyclo[2.2.2]octane unit. In addition to a simplified core structure, **1** only has one stereocenter as compared to seven in AbC. Furthermore, the benzylated derivative **2** was also envisioned to mimic the favorable toxicity profile observed for atrop-*O*-benzyl-desmethyl-abyssomicin C (Figure 1) [10]. Here we present the synthesis and biological evaluation of **1** and **2**, the hitherto most truncated derivatives of AbC.

## Results and Discussion

**Figure 2:**
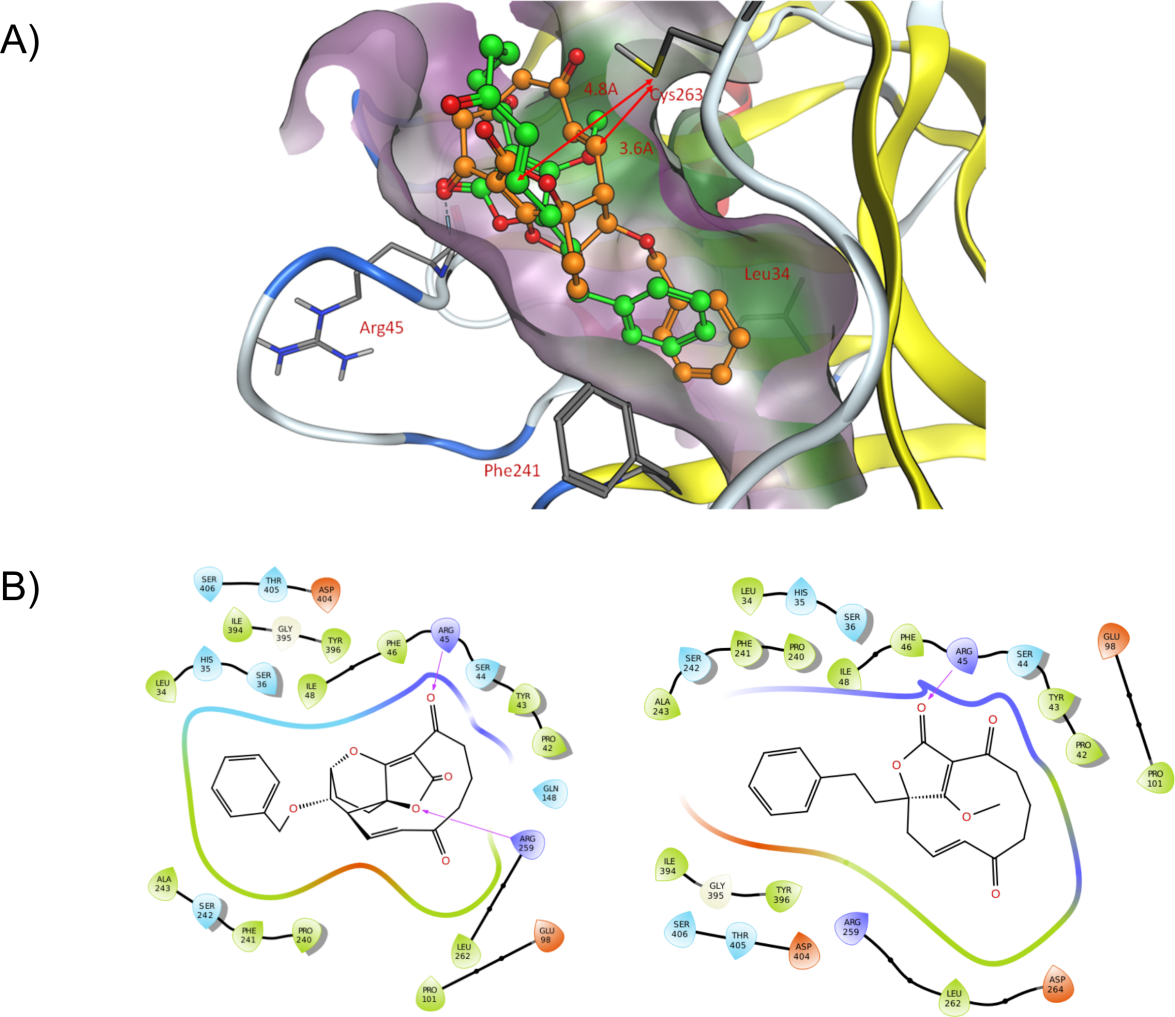
A) Docking poses for atrop-*O*-benzyl-desmethyl-abyssomicin C (orange) and compound **2** (green) showing key interactions and reasonable positioning for a reaction with Cys263. B) Ligand interaction diagrams for atrop-*O*-benzyl-desmethyl-abyssomicin C (left) and compound **2** (right), showing similar interactions and similar shape.

An initial evaluation of the truncation strategy was made by assessing the potential efficiency of binding to the target by modeling. Accordingly, the crystal structure of ADC synthase (PDB ID: 1K0E) [19] was used for docking of known and proposed ligands. The crystal structure contains a tryptophan molecule in the active site. Restrained molecular dynamics [20–21] was employed to position the active site cysteine (Cys-263) in a position that would allow covalent binding of the ligands in the active site. The resulting protein conformation was allowed to relax to ensure that the found conformation was an energy minimum. This protein structure was then used for docking of ligands using Glide [22–24]. Glide XP docking positioned both AbC and the analog atrop-*O*-benzyl-desmethyl-abyssomicin C with low energy conformations and with expected interaction points in the binding site, providing suitable docking scores (see Supporting Information File 1). The key interactions include a hydrogen bond to the backbone of Arg-45 and lipophilic interactions in the deep pocket defined by Phe-241 and Leu-34 (Figure 2). Our compound **2** docks in a similar way, filling the same subpocket, maintaining the key interactions and positioning the Cys-263 near the reacting double bond (Figure 2A). The additional ring system of atrop-*O*-benzyl-desmethyl-abyssomicin C does not seem to make any significant interaction with the protein, which suggests a suitable level of structural truncation of compound **2**.

Further docking studies, using covalent docking, also showed that both atrop-*O*-benzyl-desmethyl-abyssomicin C and **2** can bind to the active site cysteine via a Michael addition to the α,β-unsaturated ketone, still maintaining the benzyl group in the favorable subpocket position (Figure S1 in Supporting Information File 1).

### Synthesis

**Scheme 1:**
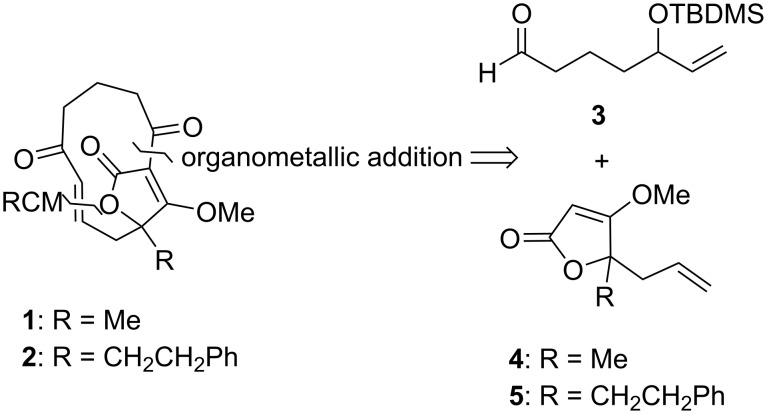
Retrosynthetic analysis of the truncated derivatives **1** and **2** of abyssomicin C.

The syntheses of **1** and **2** were inspired, in part, by previous strategies employed in the total synthesis of AbC [7–9,16–17]. Hence, the construction of the 11-membered ring was based on a carbonyl addition reaction between aldehyde **3** and the tetronate derivatives **4** and **5**, followed by a ring-closing metathesis (Scheme 1). Since both enantiomers of AbC have similar activity [25], we pursued racemic synthesis of the targeted compounds.

**Scheme 2:**
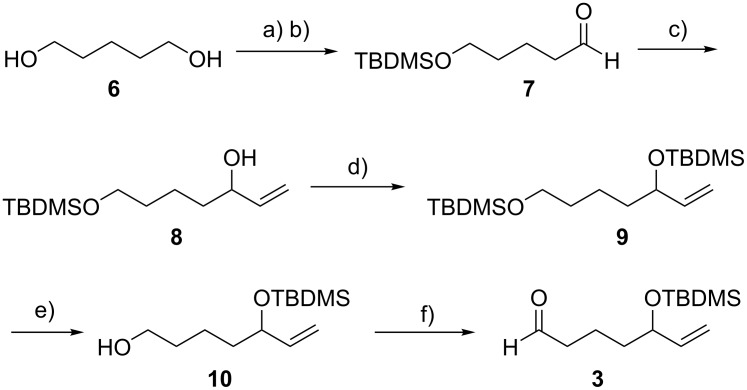
Synthesis of the building block **3**. a) TBDMSCl (0.5 equiv), NaH (1.0 equiv), THF, 0 °C to rt, 16 h, 82%. b) i: (COCl)_2_ (1.2 equiv), DMSO (2.4 equiv), CH_2_Cl_2_, −78 °C, 1.5 h; ii: Et_3_N (5.0 equiv), −78 °C to rt, 1 h, 70%. c) vinylmagnesium bromide (1.4 equiv), CeCl_3_ (1.4 equiv), THF, −78 °C, 2 h, then rt, 30 min, 89%. d) TBDMSCl (2.0 equiv), imidazole (4.0 equiv), DMF, rt, 2 h, 95%. e) HF·pyridine, pyridine, THF, rt, 45 h, 75%. f) i: (COCl)_2_ (1.2 equiv), DMSO (2.4 equiv), CH_2_Cl_2_, −78 °C, 1.5 h; ii: Et_3_N (5.0 equiv), −78 °C to rt, 1 h, 91%.

We synthesized the common building block **3** in six steps (Scheme 2), starting with the monoprotection of 1,5-pentanediol (**6**), followed by Swern oxidation, which afforded **7** in an overall yield of 57% [26]. The so obtained aldehyde **7** was treated with vinylmagnesium bromide in the presence of cerium chloride to afford **8** in 89% yield. An overall translocation of the TBDMS group from the primary to the secondary alcohol was achieved by a protection/deprotection sequence, which smoothly provided alcohol **10** in 71% yield over two steps. A final Swern oxidation gave the building block **3** in 91% yield (33% overall yield from 1,5-pentanediol **6**).

**Scheme 3:**
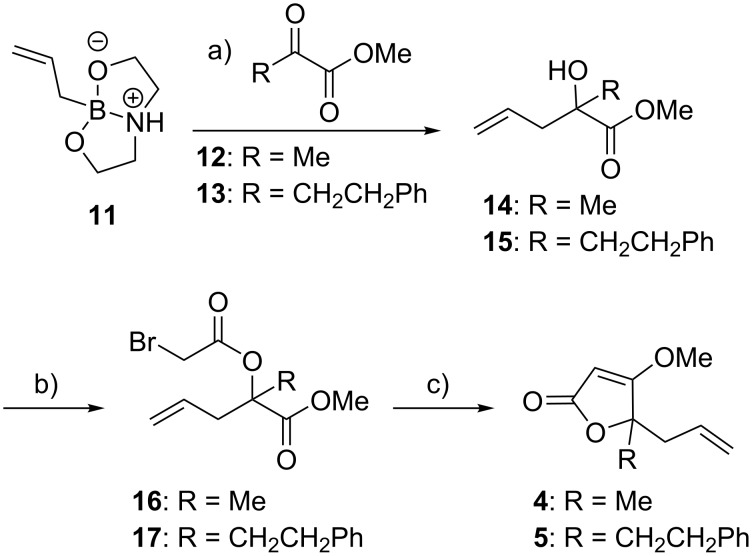
Synthesis of the building blocks **4** and **5**. a) TFA (1.1 equiv), CH_2_Cl_2_, rt, 24 h, 84% (**14**: R = Me) and 92% (**15**: R = CH_2_CH_2_Ph). b) bromoacetyl bromide (1.5–2.4 equiv), toluene, reflux, 20–24 h, 68% (**16**: R = Me) and 72% (**17**: R = CH_2_CH_2_Ph). c) PPh_3_ (1.5 equiv), DIPEA (1.2 equiv), THF, 70 °C, 16 h, 75% (**4**: R = Me) and 77% (**5**: R = CH_2_CH_2_Ph).

The other key intermediates, building blocks **4** and **5** (**4**, R = Me and **5**, R = CH_2_CH_2_Ph) were synthesized starting with allyldioxazaborolidine **11**, an allyl-transfer reagent that was prepared as previously reported (Scheme 3) [27]. Allylation of methyl pyruvate (**12**) or **13** (synthesized from dimethyl oxalate and phenethylmagnesium bromide, see Supporting Information File 1) using **11** in the presence of trifluoroacetic acid afforded the corresponding homoallylic alcohol **14** and **15** in 84% and 92% yield, respectively. The next steps involved acylation of alcohol **14** and **15** using bromoacetyl bromide in refluxing toluene, which afforded the corresponding α-bromo esters **16** and **17**, in 68% and 72% yield, respectively. Finally, intramolecular Wittig reaction of **16** or **17** in the presence of *N*,*N*-diisopropylethylamine and triphenylphosphine provided building blocks **4** and **5** in 75% and 77% yield (43% and 51% overall yields from allyldioxazaborolidine **11**), respectively.

**Scheme 4:**
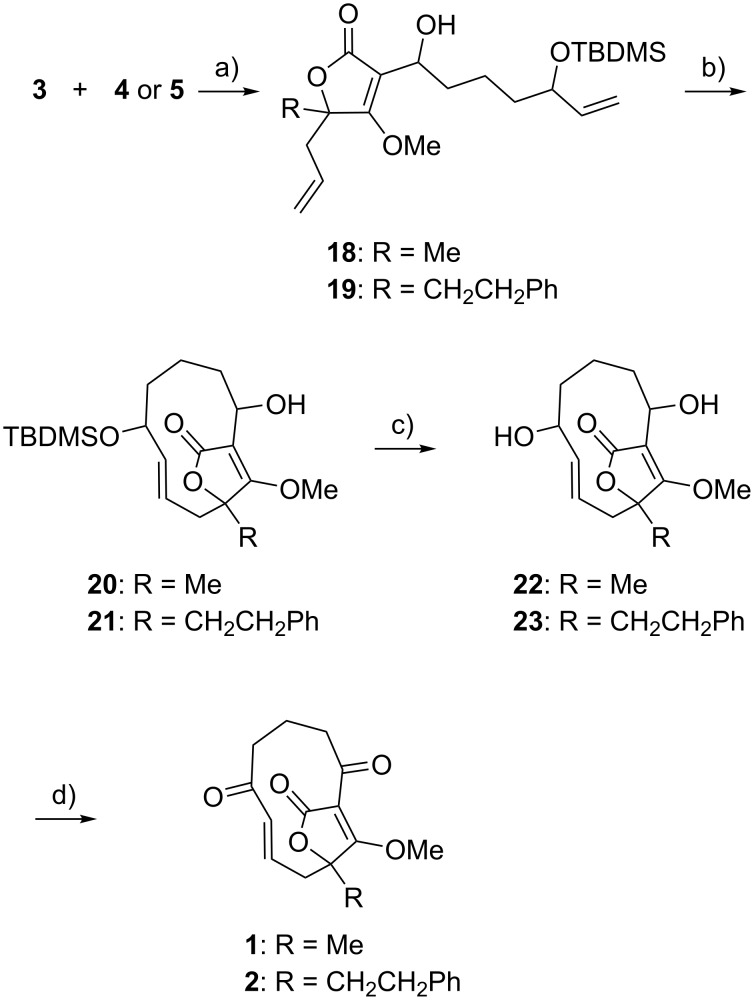
Reaction of the building block **3** with **4** or **5** for the synthesis of compounds **1** and **2**. a) **4** or **5** with LDA (1.5 equiv), THF, −78 °C, 30 min; then **3**, −78 °C, 1.5 h; 78% (**18**: R = Me) and 59% (**19**: R = CH_2_CH_2_Ph). b) Hoveyda–Grubbs second generation catalyst (5 mol %), 1,2-dichloroethane (0.002 M), reflux, 1 h, 62% (**20**: R = Me) and 45% (**21**: R = CH_2_CH_2_Ph). c) TBAF (10 equiv), THF, rt, 16 h, 73% (**22**: R = Me) and 74% (**23**: R = CH_2_CH_2_Ph). d) Dess–Martin periodinane (2.5 equiv), CH_2_Cl_2_, rt, 2 h, 24% (**1**: R = Me) and 41% (**2**: R = CH_2_CH_2_Ph).

The tetronate building blocks **4** and **5** were further converted to the corresponding enolates using lithium diisopropylamide and allowed to react with the common key intermediate **3** to afford the hydroxyalkylated products **18** and **19**, in 78% and 59% yield, respectively (Scheme 4). Treatment of **18** and **19** with Hoveyda–Grubbs second generation catalyst in refluxing 1,2-dichloroethane afforded the ring-closing metathesis products **20** and **21** in 62% and 45% yield, respectively. Deprotection of the TBDMS group using tetrabutylammonium fluoride followed by final Dess–Martin oxidation gave the targeted compounds **1** and **2** in 17% and 30% yield over the two steps, respectively. For the final step, several oxidation agents were evaluated, and the highest yields were achieved with Dess–Martin periodinane, which converted **22** and **23** to **1** and **2** in 24% and 41% yield, respectively.

### Biological evaluation

The antibacterial activity of the truncated AbC derivatives **1** and **2**, as well as the tetronate building blocks **4** and **5**, was evaluated against *S. aureus* and two different strains of MRSA using vancomycin as a positive control (Table 1).

**Table 1:** Minimum inhibitory concentrations (MIC) of compounds **1**, **2**, **4** and **5** against *S. aureus* and two strains of MRSA.

Compound	MIC (µg/mL)		

	*S. aureus* CCUG15915	MRSA CCUG58065	MRSA CCUG38266

vancomycin	1	1	0.5
**1**	>200	>200	>200
**2**	150	150	100
**4**	>200	>200	>200
**5**	>200	>200	>200

It has been previously shown that structural prerequisites for truncated derivatives of AbC to harbor activity against Gram-positive bacteria are that the 11-membered ring is intact and that this macrocycle is equipped with an enone functionality [4–5]. In accordance with these previous findings, compounds **4** and **5** were found not to inhibit the growth of any of the bacterial strains investigated. Likewise, and to our dismay, the targeted AbC derivative **1** did not show any inhibition either. Only compound **2** showed a low inhibition of bacterial growth (100–150 μg/mL), which, taken together, suggests that the oxabicyclo[2.2.2]octane moiety from the original structure of AbC is crucial for antibacterial activity. The activity difference between compounds **1** and **2** could be explained by the predicted more efficient binding of **2** with the active site. However, such a conclusion would be highly speculative based solely on these results. Due to the low activity of compound **2**, no further investigations related to the conserved mode of action or toxicity were performed.

## Conclusion

We have described the synthesis and antibacterial activity of two new truncated derivatives of AbC. Previous work indicated that the three methyl groups of AbC were not required for activity [10]. In this work, we went one step further with the design of two new truncated AbC derivatives, in which besides removing the three methyl groups, the oxabicyclo[2.2.2]octane structure was also subjected to truncation. We found that the antibacterial activity is either decreased by one order of magnitude or even completely lost, given that the MIC values of the two new derivatives are ≥100 µg/mL. These unexpectedly low activities could possibly be rationalized from the initial modeling studies, which predicted a longer distance from the reacting cysteine to the electrophilic carbon associated with the enone functionality for compound **2** than for the active atrop-*O*-benzyl-desmethyl-abyssomicin C (Figure 2A). This difference is not reflected in the docking scores, however, it is still indicative of a less favorable binding for **2** when compared to atrop-*O*-benzyl-desmethyl-abyssomicin C. Even though these results are somewhat discouraging, together with previous studies they clearly outline the upper level of truncation that is tolerated for further SAR studies of AbC derivatives. As such, this work demonstrates that continued efforts towards drug development based on AbC should focus on derivatives that include structural motifs close to the oxabicyclo[2.2.2]octane with substituents that can more systematically explore interactions with the deep channel of the ADC synthase.

## Supporting Information

File 1 Experimental part (modelling and docking, synthesis and biological evaluation), and copies of NMR spectra.
