# Supplementary material for: Synthesis and biological evaluation of truncated derivatives of abyssomicin C as antibacterial agents
Source: Beilstein J Org Chem. 2019 Jul 2;15:1468–74. doi: 10.3762/bjoc.15.147 (PMC6633193; doi:10.3762/bjoc.15.147)
Supplement: File 1 — Experimental part (modelling and docking, synthesis and biological evaluation), and copies of NMR spectra. [file Beilstein_J_Org_Chem-15-1468-s001.pdf]

BEILSTEIN JOURNAL OF ORGANIC CHEMISTRY

Supporting Information

for

Synthesis and biological evaluation of truncated derivatives of abyssomicin C as antibacterial agents

Leticia Monjas, Peter Fodran, Johanna Kollback, Carlo Cassani, Thomas Olsson, Maja Genheden, D. G. Joakim Larsson and Carl-Johan Wallentin

Beilstein J. Org. Chem. **2019,** *15,* 1468–1474. doi:10.3762/bjoc.15.147

Experimental part (modelling and docking, synthesis and biological evaluation), and copies of NMR spectra

License and Terms: This is a supporting information file under the terms of the Creative Commons Attribution License (http://creativecommons.org/licenses/by/4.0). Please note that the reuse, redistribution and reproduction in particular requires that the authors and source are credited.
The license is subject to the *Beilstein Journal of Organic Chemistry* terms and conditions: (https://www.beilstein-journals.org/bjoc)

List of contents

1. Experimental part S2

1.1 Modeling and docking S2

1.2 Synthesis S3

1.3 Biological evaluation: antibacterial activity S11

2. References S11

3. NMR spectra S12

1. Experimental part

1.1 Modeling and docking

General. The crystal structure of 4-amino4-deoxychorismate (ADC) synthase (PDB ID: 1K0E) [1] was used for modeling and docking. Abyssomicin C (AbC) was docked in this protein using the tryptophan molecule in the crystal structure to define the binding pocket.

Molecular dynamics (MD). It is known that AbC binds covalently to the Cys-263 in ADC synthase. In the crystal structure this cysteine is positioned too far away from the ligand to be able to react. Therefore, as a first step, AbC was docked in the 1K0E structure using Glide in order to obtain a reasonable starting position for ligands. Restrained MD using Gromacs [2,3] was then used to investigate if Cys-263 could be located in a position where a Michael reaction with AbC was possible. A distance restraint of 4.5 Å between the sulfur of Cys-263 and the β-carbon of the α,β-unsaturated ketone of AbC was used, which resulted in a protein conformation that has a suitable geometry for covalent binding of the ligand. The stability of the protein conformation obtained by this restriction was verified by removing the restraint in a subsequent MD run. This proved the restrained conformation to be stable. The structure obtained by restrained MD was used in all further docking.

Covalent docking. The covalent docking module in the Schrödinger package was used to covalently dock ligands [4–6]. The sulfur of Cys-263 was selected as nucleophile and a 1,4 addition (Michael reaction) was selected to obtain the covalent binding. This resulted in good docked positions for AbC, atrop-AbC, *O*-benzyl-desmethylabyssomicin C, atrop-*O*-benzyl-desmethylabyssomicin C as well as for the truncated derivative **2**.

Compound	Covalent docking score (kcal/mol)
abyssomicin C	−4.13
atrop-abyssomicin C	−5.05
O-benzyl-desmethylabyssomicin C	−6.46
atrop-*O*-benzyl-desmethylabyssomicin C	−5.71
compound **2**	−6.63

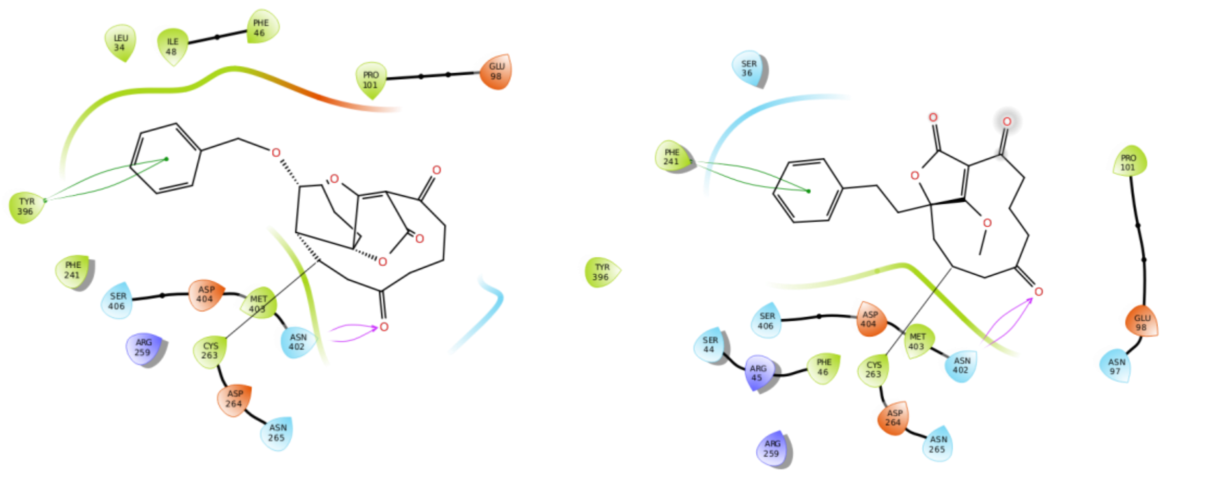

Figure S1: Ligand interaction diagram for atrop-*O*-benzyl-desmethyl-abyssomicin C (left) and compound **2** (right) from covalent docking, showing similar interactions and similar shape

Glide docking. Glide XP docking was performed in order to investigate the ability for the different ligands to bind in the active site of the protein, before a reaction with Cys-263. Positioning the ligand in an appropriate position is required for covalent binding of the ligand. Compound **1** gives significantly lower scores and no distinct docking pose and is therefore omitted from the tables.

Compound	Glide XP docking score (kcal/mol)
abyssomicin C	–4.91
atrop-abyssomicin C	–5.43
O-benzyl-desmethylabyssomicin C	–8.37
atrop-*O*-benzyl-desmethylabyssomicin C	–9.22
compound **2**	–6.46

1.2 Synthesis

General methods. All reagents were purchased from Sigma-Aldrich, Fluorochem and VWR Chemicals and were used without further purification unless noted otherwise. Air sensitive reactions were performed using oven-dried glassware (T_{oven} = 150 °C) and performed under a nitrogen atmosphere using Schlenk techniques. Solvents were dried on a solvent purification system (PS-MD-5/7 Inert technology). Reactions were monitored either by GC–MS (GC System 7820A, MSD 5977E Agilent Technologies) using the following temperature program: T_0 = 60 °C, hold 1 min; ramp 40 °C min^{-1} to 320 °C, hold 1 min; or by thin-layer chromatography (TLC) on silica-gel-coated aluminum foils (silica gel 60 F254, Merck). The TLC plates were visualized by UV light (λ = 254 nm) or by staining with the following solution: $KMnO_4$ (2.0 g), K_2CO_3 (14.0 g), NaOH (0.2 g) in water (200 mL) and heating. Flash-column chromatography was performed on silica gel (VWR Chemicals, 40–63 µm). NMR spectra were recorded on a Varian NMR 400 spectrometer at 25 °C. Chemical shifts (δ) are reported in ppm relative to the residual solvent peak. Splitting patterns are indicated as (s) singlet, (d) doublet, (t) triplet, (q) quartet, (m) multiplet and (br) broad. Coupling constants (*J*) are reported in Hertz (Hz). High-resolution mass spectra (HRMS) were recorded on a Waters XEVO-G2 QTOF mass spectrometer.

Compound **7** was synthesized according to a reported procedure and its spectroscopic data are in agreement [7].

7-((*tert*-Butyldimethylsilyl)oxy)hept-1-en-3-ol (8):

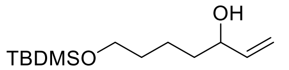

A round-bottom flask charged with $CeCl_3 \cdot 7H_2O$ (*ca.* 50 g) was heated to 200 °C under vacuum for 48 h. The dried $CeCl_3$ (24.6 g, 100 mmol) was suspended in anhydrous THF (350 mL) at –78 °C under a nitrogen atmosphere. Vinylmagnesium bromide (1 M in THF, 100 mL, 100 mmol) was added dropwise and the reaction mixture was stirred at –78 °C for 1.5 h. Then, **7** (15.2 g, 70.2 mmol, 0.70 equiv) was added dropwise, and the reaction mixture was stirred at –78 °C for 2 h. Then, the reaction was allowed to warm up to room temperature, stirred for 30 min, and quenched with an aqueous solution of acetic acid (10% v/v, 500 mL). Et_2O (400 mL) was added and the layers were separated. The aqueous layer was extracted with Et_2O (2 × 200 mL), and the combined organic layers were washed with an aqueous saturated solution of $NaHCO_3$ and brine (1:1, 1 × 400 mL), brine (1 × 400 mL), dried over Na_2SO_4, filtered and concentrated in vacuo. Compound **8** was obtained as a pale yellow oil (15.3 g, 62.4 mmol, 89%) and used without further purification. ^{1}H-NMR (400 MHz, $CDCl_3$) δ 5.87 (ddd, *J* = 17.2, 10.4, 6.2 Hz, 1H), 5.22 (dt, *J* = 17.2, 1.4 Hz, 1H), 5.10 (ddd, *J* = 10.4, 1.4, 1.1 Hz, 1H), 4.13–4.08 (m, 1H), 3.61 (t, *J* = 6.5 Hz, 2H), 1.73–1.69 (m, 1H), 1.59–1.38 (m, 6H), 0.89 (s, 9H), 0.04 (s,

6H). ^{13}C-NMR (101 MHz, $CDCl_3$) δ 141.3, 114.8, 73.3, 63.2, 36.9, 32.8, 26.1, 21.8, 18.5, –5.1. The spectroscopic data are in agreement with those reported in the literature [8,9].

2,2′,3,3′,11,11′,12,12′-Octamethyl-5-vinyl-4,10-dioxa-3,11-disilatridecane (9):

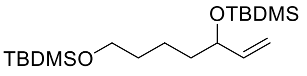

To a solution of **8** (15.2 g, 62.1 mmol) in DMF (63 mL), TBDMSCl (18.7 g, 124.3 mmol, 2.0 equiv) and imidazole (16.9 g, 248.6 mmol, 4.0 equiv) were added. The reaction mixture was stirred at room temperature for 2 h. Then, water (500 mL) and Et_2O (300 mL) were added and the layers were separated. The aqueous layer was extracted with Et_2O (2 × 200 mL), and the combined organic layers were washed with brine (1 × 200 mL), dried over Na_2SO_4, filtered and concentrated in vacuo. Purification by flash column chromatography (hexane:MTBE 98:2) afforded **9** as a colorless oil (21.1 g, 58.8 mmol, 95%). ^{1}H-NMR (400 MHz, $CDCl_3$) δ 5.79 (ddd, J = 17.2, 10.4, 6.1 Hz, 1H), 5.12 (ddd, J = 17.2, 1.9, 1.3 Hz, 1H), 5.01 (ddd, J = 10.4, 1.9, 1.2 Hz, 1H), 4.10–4.05 (m, 1H), 3.60 (t, J = 6.5 Hz, 2H), 1.56–1.26 (m, 6H), 0.89 (m, 18H), 0.05–0.03 (m, 12H). ^{13}C-NMR (101 MHz, $CDCl_3$) δ 142.0, 113.6, 74.0, 63.4, 38.1, 33.0, 26.14, 26.05, 21.7, 18.5, 18.4, –4.2, –4.7, –5.12, –5.12. The spectroscopic data are in agreement with those reported in the literature [8,9].

5-((*tert*-Butyldimethylsilyl)oxy)hept-6-en-1-ol (10):

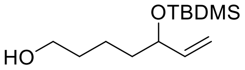

To a solution of **9** (5.7 g, 15.9 mmol) in THF (615 mL) in a polypropylene container, a solution of HF·pyridine [prepared using a commercially available solution of HF·pyridine (Sigma-Aldrich: pyridine ≈30%, HF ≈70%, 8.0 mL), which was dissolved in THF (68 mL) in a polypropylene container and diluted by adding pyridine (21.6 mL) portion wise (exothermic!)] was added at room temperature, and the reaction mixture was stirred at room temperature for 45 h. Then, the reaction was quenched by slow addition of a saturated aqueous solution of $NaHCO_3$ (400 mL) (exothermic!). The mixture was extracted with EtOAc (3 × 300 mL), and the combined organic layers were washed with brine (1 × 300 mL), dried over Na_2SO_4, filtered and concentrated in vacuo. Purification by flash column chromatography (hexane:Et_2O 7:3), afforded **10** as a colorless oil (2.93 g, 12.0 mmol, 75%). ^{1}H-NMR (400 MHz, $CDCl_3$) δ 5.79 (ddd, J = 17.2, 10.4, 6.1 Hz, 1H), 5.13 (ddd, J = 17.2, 1.9, 1.3 Hz, 1H), 5.02 (ddd, J = 10.4, 1.9, 1.2 Hz, 1H), 4.12–4.07 (m, 1H), 3.64 (t, J = 6.5 Hz, 2H), 1.61–1.32 (m, 7H, overlaps with H_2O signal), 0.89 (s, 9H), 0.05 (s, 3H), 0.03 (s, 3H). ^{13}C-NMR (101 MHz, $CDCl_3$) δ 141.8, 113.8, 73.9, 63.1, 37.9, 32.9, 26.0, 21.4, 18.4, –4.2, –4.7. The spectroscopic data are in agreement with those reported in the literature [8,9].

5-((*tert*-Butyldimethylsilyl)oxy)hept-6-enal (3):

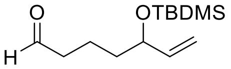

To a solution of $(COCl)_2$ (0.42 mL, 4.91 mmol, 1.2 equiv) in anhydrous CH_2Cl_2 (9 mL) at –78 °C under a nitrogen atmosphere, a solution of anhydrous DMSO (0.70 mL, 9.82 mmol, 2.4 equiv) in anhydrous CH_2Cl_2 (4 mL) was added over 10 min using a syringe pump. The reaction mixture was stirred at –78 °C for 15 min. A solution of **10** (1.0 g, 4.09 mmol, 1.0 equiv) in anhydrous CH_2Cl_2 (4 mL) was added over 10 min using a syringe pump, and the reaction mixture was stirred at –78 °C for 1 h. Then, Et_3N (2.85 mL, 20.5 mmol, 5.0 equiv) was added dropwise and the reaction mixture was stirred at –78 °C for 30 min, and then allowed to warm up to room temperature. Water (20 mL) and CH_2Cl_2 (10 mL) were added and the layers were separated. The aqueous layer was extracted with CH_2Cl_2 (3 × 10 mL), and the combined organic layers were washed with brine (1 × 15 mL), dried over Na_2SO_4, filtered and concentrated

in vacuo. Purification by flash column chromatography (hexane:Et_2O 95:5) afforded **3** as a colorless oil (0.91 g, 3.74 mmol, 91%). ^1H-NMR (400 MHz, $CDCl_3$) δ 9.76 (t, J = 1.8 Hz, 1H), 5.78 (ddd, J = 17.2, 10.4, 6.0 Hz, 1H), 5.15 (ddd, J = 17.2, 1.8, 1.3 Hz, 1H), 5.04 (ddd, J = 10.4, 1.8, 1.2 Hz, 1H), 4.14–4.09 (m, 1H), 2.44 (td, J = 7.3, 1.8 Hz, 2H), 1.74–1.48 (m, 4H, overlaps with H_2O signal), 0.89 (s, 9H), 0.05 (s, 3H), 0.03 (s, 3H). ^{13}C-NMR (101 MHz, $CDCl_3$) δ 202.8, 141.4, 114.2, 73.5, 44.0, 37.4, 26.0, 18.4, 17.9, −4.2, −4.7. HRMS (ESI+) calculated for $C_{13}H_{26}O_2SiNa$ $[M + Na]^+$ 265.1600, found 265.1595.

Allyldioxazaborolidine (11):

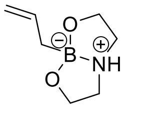

Compound **11** was synthesized as previously described,[10] with some minor modifications: CH_2Cl_2 was used for extraction during the work-up, and the compound was purified by trituration of the crude (white sticky solid) with Et_2O. The spectroscopic data are consistent with those previously reported [10].

Methyl 2-hydroxy-2-methylpent-4-enoate (14):

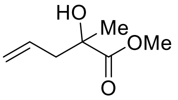

To a solution of **11** (6.78 g, 43.7 mmol, 1.1 equiv) in CH_2Cl_2 (87 mL) at room temperature, methyl pyruvate (3.6 mL, 39.8 mmol, 1.0 equiv) was added dropwise. Then, TFA (3.4 mL, 43.7 mmol, 1.1 equiv) was added dropwise and the reaction mixture was stirred at room temperature for 24 h. Then, the reaction mixture was diluted with CH_2Cl_2 (50 mL) and water (150 mL) and the layers were separated. The aqueous layer was extracted with CH_2Cl_2 (2 × 50 mL), the combined organic layers were washed with a saturated aqueous solution of $NaHCO_3$ (2 × 100 mL), brine (1 × 150 mL), dried over Na_2SO_4, filtered and concentrated in vacuo. **14** was obtained as a colorless oil (4.80 g, 33.3 mmol, 84%) and used without further purification. ^1H-NMR (400 MHz, $CDCl_3$) δ 5.76 (ddt, J = 16.5, 10.6, 7.3 Hz, 1H), 5.13–5.08 (m, 2H), 3.76 (s, 3H), 2.49 (ddt, J = 13.7, 7.3, 1.1 Hz, 1H), 2.39 (ddt, J = 13.7, 7.3, 1.1 Hz, 1H), 1.42 (s, 3H). ^{13}C-NMR (101 MHz, $CDCl_3$) δ 177.1, 132.5, 119.3, 74.6, 52.8, 44.8, 25.6. HRMS (ESI+) calculated for $C_7H_{12}O_3Na$ $[M + Na]^+$ 167.0684, found 167.0681.

Methyl 2-(2-bromoacetoxy)-2-methylpent-4-enoate (16):

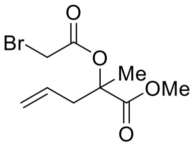

To a solution of **14** (4.50 g, 31.2 mmol) in toluene (35 mL), bromoacetyl bromide (4.08 mL, 46.8 mmol, 1.5 equiv) was added. The reaction mixture was stirred at reflux (130 °C, pre-heated oil bath) for 20 h. Then, the reaction was allowed to cool down, MeOH (30 mL) was added and the mixture was stirred for 5 min at room temperature. Then, the solvent was evaporated in vacuo. Purification by flash column chromatography (hexane:Et_2O 97:3), afforded **16** as a pale yellow oil (5.62 g, 21.2 mmol, 68%). ^1H-NMR (400 MHz, $CDCl_3$) δ 5.75 (ddt, J = 16.9, 10.3, 7.3 Hz, 1H), 5.19–5.11 (m, 2H), 3.84 (s, 2H), 3.74 (s, 3H), 2.75 (ddt, J = 14.2, 7.3, 1.2 Hz, 1H), 2.57 (ddt, J = 14.2, 7.3, 1.2 Hz, 1H), 1.59 (s, 3H). ^{13}C-NMR (101 MHz, $CDCl_3$) δ 171.7, 166.1, 130.9, 120.1, 82.1, 52.7, 42.1, 26.0, 21.4. HRMS (ESI+) calculated for $C_9H_{13}O_4BrNa$ $[M + Na]^+$ 286.9895, found 286.9892.

5-Allyl-4-methoxy-5-methylfuran-2(5*H*)-one (4):

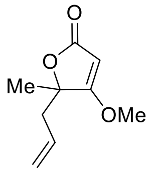

To a solution of **16** (5.40 g, 20.4 mmol) in THF (82 mL), PPh_3 (8.01 g, 30.6 mmol, 1.5 equiv) and DIPEA (4.3 mL, 24.4 mmol, 1.2 equiv) were added sequentially. The reaction mixture was stirred at 70 °C (pre-heated oil bath) for 16 h. Then, the reaction was cooled down to 0 °C, filtered over celite to remove the precipitate, and the solvent was evaporated in vacuo. Purification by flash column chromatography (hexane:EtOAc 4:1) afforded **4** as a pale yellow oil (2.58 g, 15.3 mmol, 75%). ^{1}H-NMR (400 MHz, $CDCl_3$) δ 5.72–5.61 (m, 1H), 5.13–5.08 (m, 2H), 4.99 (s, 1H), 3.86 (s, 3H), 2.54 (ddt, *J* = 14.2, 7.3, 1.1 Hz, 1H), 2.42 (ddt, *J* = 14.2, 7.3, 1.1 Hz, 1H), 1.45 (s, 3H). ^{13}C-NMR (101 MHz, $CDCl_3$) δ 184.7, 171.8, 130.9, 119.8, 88.4, 84.0, 59.5, 41.4, 23.1. HRMS (ESI+) calculated for $C_9H_{12}O_3Na$ $[M + Na]^+$ 191.0684, found 191.0681.

Methyl 2-oxo-4-phenylbutanoate (13):

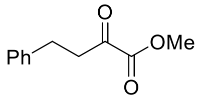

To a solution of dimethyl oxalate (1.71 g,14.5 mmol, 1.1 equiv) in anhydrous THF (29 mL) at −78 °C under a nitrogen atmosphere, freshly prepared phenethylmagnesium bromide (titrated, 0.6 M in Et_2O, 22 mL, 13.2 mmol, 1.0 equiv) was added dropwise over 1 h using a syringe pump. The reaction mixture was stirred at −78 °C for 1 h, and then allowed to warm up to room temperature. The reaction was quenched with aqueous HCl (3 M, 30 mL) and water (30 mL). Et_2O (70 mL) was added and the layers were separated. The aqueous layer was extracted with Et_2O (4 × 30 mL), and the combined organic layers were washed with brine (1 × 100 mL), dried over Na_2SO_4, filtered and concentrated in vacuo. Purification by flash column chromatography (hexane:Et_2O 85:15) afforded **13** as a pale yellow oil (2.34 g, 12.2 mmol, 92%). ^{1}H-NMR (400 MHz, $CDCl_3$) δ 7.31–7.27 (m, 2H), 7.23–7.19 (m, 3H), 3.86 (s, 3H), 3.19 (t, *J* = 7.5 Hz, 2H), 2.97 (t, *J* = 7.5 Hz, 2H). ^{13}C-NMR (101 MHz, $CDCl_3$) δ 193.3, 161.4, 140.1, 128.7, 128.5, 126.5, 53.1, 41.1, 29.1. The spectroscopic data are in agreement with those reported in the literature [11].

Methyl 2-hydroxy-2-phenethylpent-4-enoate (15):

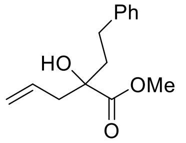

To a solution of **11** (2.03 g, 13.1 mmol, 1.1 equiv) in CH_2Cl_2 (30 mL) at room temperature, **13** (2.29 g, 11.9 mmol, 1.0 equiv) was added dropwise. Then, TFA (1.0 mL, 13.1 mmol, 1.1 equiv) was added dropwise and the reaction mixture was stirred at room temperature for 24 h. Then, the reaction mixture was diluted with CH_2Cl_2 (80 mL) and water (50 mL) and the layers were separated. The aqueous layer was extracted with CH_2Cl_2 (3 × 30 mL), the combined organic layers were washed with a saturated aqueous solution of $NaHCO_3$ (1 × 80 mL), brine (1 × 80 mL), dried over Na_2SO_4, filtered and concentrated in vacuo. **15** was obtained as a colorless oil (2.58 g, 11.0 mmol, 92%) and used without further purification. ^{1}H-NMR (400 MHz, $CDCl_3$) δ 7.29–7.25 (m, 2H, overlaps with $CDCl_3$ signal), 7.20–7.16 (m, 3H), 5.82–5.72 (m, 1H), 5.13–5.08 (m, 2H), 3.72 (s, 3H), 2.85–2.78 (m, 1H), 2.52–2.39 (m, 3H), 2.13–1.96 (m, 2H). ^{13}C-NMR (101 MHz, $CDCl_3$) δ 176.5, 141.6, 132.3, 128.6, 128.5, 126.1, 119.3, 52.9, 44.3, 40.6, 30.1. A quaternary C overlaps with the $CDCl_3$ signal. HRMS (ESI+) calculated for $C_{14}H_{18}O_3Na$ $[M + Na]^+$ 257.1154, found 257.1157.

Methyl 2-(2-bromoacetoxy)-2-phenethylpent-4-enoate (17):

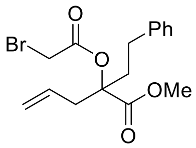

To a solution of **15** (2.58 g, 11.0 mmol) in toluene (20 mL), bromoacetyl bromide (3.2 mL, 26.4 mmol, 2.4 equiv) was added in two portions (the second one after 16 h of reaction time). The reaction mixture was stirred at reflux (130 °C, pre-heated oil bath) for 24 h. Then, the reaction was allowed to cool down, MeOH (10 mL) was added and the mixture was stirred for 5 min at room temperature. Then, the solvent was evaporated in vacuo. Purification by flash column chromatography (hexane:Et_2O from 97:3 to 95:5), afforded **17** as a pale yellow oil (2.81 g, 7.92 mmol, 72%). ^{1}H-NMR (400 MHz, $CDCl_3$) δ 7.30–7.26 (m, 2H), 7.22–7.15 (m, 3H), 5.74 (ddt, *J* = 16.0, 11.0, 7.4 Hz, 1H), 5.19–5.14 (m, 2H), 3.80 (s, 2H), 3.74 (s, 3H), 2.97 (ddt, *J* = 14.7, 7.4, 1.2 Hz, 1H), 2.78 (ddt, *J* = 14.7, 7.4, 1.2 Hz, 1H), 2.73–2.55 (m, 2H), 2.36–2.21 (m, 2H). ^{13}C-NMR (101 MHz, $CDCl_3$) δ 171.1, 166.0, 140.9, 130.9, 128.6, 128.5, 126.3, 120.1, 84.3, 52.6, 38.6, 36.1, 29.5, 25.8. HRMS (ESI+) calculated for $C_{16}H_{19}O_4BrNa$ $[M + Na]^+$ 377.0364, found 377.0356.

5-Allyl-4-methoxy-5-phenethylfuran-2(5*H*)-one (5):

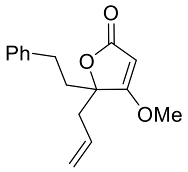

To a solution of **17** (2.54 g, 7.15 mmol) in THF (30 mL), PPh_3 (2.81 g, 10.7 mmol, 1.5 equiv) and DIPEA (1.5 mL, 8.58 mmol, 1.2 equiv) were added. The reaction mixture was stirred at 70 °C (pre-heated oil bath) for 16 h. Then, the reaction was cooled down to 0 °C and filtered over celite to remove the precipitate, and the solvent was evaporated in vacuo. Purification by flash column chromatography (hexane:EtOAc 4:1) afforded **5** as a white thick oil (1.43 g, 5.51 mmol, 77%). ^{1}H-NMR (400 MHz, $CDCl_3$) δ 7.29–7.24 (m, 2H, overlaps with $CDCl_3$ signal), 7.20–7.13 (m, 3H), 5.72–5.62 (m, 1H), 5.14–5.09 (m, 2H), 5.04 (s, 1H), 3.78 (s, 3H), 2.71–2.63 (m, 1H), 2.60 (ddt, *J* = 14.2, 7.0, 1.2 Hz, 1H). 2.48–2.40 (m, 2H), 2.18–1.99 (m, 2H). ^{13}C-NMR (101 MHz, $CDCl_3$) δ 182.9, 171.9, 140.9, 130.5, 128.54, 128.53, 126.3, 120.0, 90.0, 86.1, 59.4, 40.9, 37.2, 29.2. HRMS (ESI+) calculated for $C_{16}H_{18}O_3Na$ $[M + Na]^+$ 281.1154, found 281.1147.

5-Allyl-3-(5-((*tert*-butyldimethylsilyl)oxy)-1-hydroxyhept-6-en-1-yl)-4-methoxy-5-methylfuran-2(5*H*)-one (18):

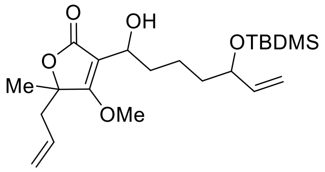

To a solution of iPr_2NH (filtered over a short column of basic Al_2O_3 just before the reaction, 0.70 mL, 5.01 mmol, 1.5 equiv) in anhydrous THF (8 mL) at −78 °C under a nitrogen atmosphere, *n*-BuLi (2.5 M in hexanes, 2.0 mL, 5.01 mmol, 1.5 equiv) was added. The reaction mixture was stirred at −78 °C for 30 min. A solution of **4** (561 mg, 3.34 mmol, 1.0 equiv) in anhydrous THF (1 mL) was added and the reaction mixture was stirred at −78 °C for 30 min. Then, a solution of **3** (890 mg, 3.67 mmol, 1.1 equiv) in anhydrous THF (3 mL) was added and the reaction mixture was stirred at −78 °C for 1.5 h, and then it was allowed to warm up to room temperature. The reaction was quenched with water (20 mL) and extracted with EtOAc (3 × 10 mL). The combined organic layers were dried over Na_2SO_4, filtered and concentrated in vacuo. Purification by flash column chromatography (hexane:EtOAc 85:15) afforded **18** as a yellow oil (1.06 g, 2.59 mmol, 78%). ^{1}H-NMR (400 MHz, $CDCl_3$) δ 5.80–5.54 (m, 2H), 5.14–5.06 (m, 3H), 5.02–4.98 (m, 1H), 4.74–4.69 (m, 1H), 4.09–4.04 (m, 4H), 3.49 (br s, 1H), 2.53–2.35 (m, 2H), 1.92–1.84 (m, 1H), 1.74–1.68 (m, 1H), 1.53–1.26 (m, 7H), 0.87 (m, 9H), 0.03–0.01 (m, 6H). ^{13}C-NMR (101 MHz, $CDCl_3$) δ 175.0, 174.43, 174.41, 173.05, 173.04, 141.69, 141.67, 141.63, 130.8, 119.89, 119.88, 113.93, 113.92, 113.90, 113.86, 103.9, 103.74, 103.71, 83.3, 83.2, 73.93, 73.83, 73.78, 73.71, 66.69, 66.60, 66.55, 66.4, 60.2, 60.1, 41.35, 41.33, 38.2, 38.05, 38.03,

37.93, 37.88, 37.87, 37.82, 29.8, 26.0, 23.2, 23.0, 22.14, 22.05, 22.04, 21.9, 18.36, 18.35, −4.23, −4.26, −4.69, −4.71, −4.72. The NMR shifts of all diastereomers are reported. HRMS (ESI+) calculated for $C_{22}H_{38}O_5SiNa$ [M + Na]$^+$ 433.2386, found 433.2379.

(1*E*,7*E*)-6-((*tert*-Butyldimethylsilyl)oxy)-2-hydroxy-13-methoxy-10-methyl-11-oxabicyclo[8.2.1]trideca-1(13),7-dien-12-one (20):

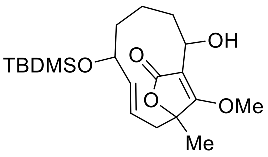

To a solution of **18** (250 mg, 0.609 mmol) in 1,2-dichloroethane (294 mL) at 103 °C (pre-heated oil bath), a solution of Hoveyda–Grubbs second generation catalyst (19.0 mg, 30.4 µmol, 5 mol %) in 1,2-dichloroethane (10 mL) was added (the final concentration of substrate was 0.002 M). The reaction mixture was stirred at reflux for 1 h. Then, the reaction was cooled to room temperature and the solvent evaporated in vacuo. Purification by flash column chromatography (hexane: EtOAc 4:1) afforded **20** as a pale brown oil (145 mg, 0.378 mmol, 62%). ^{1}H-NMR (400 MHz, $CDCl_3$) δ 5.58–5.19 (m, 2H), 4.66–4.49 (m, 1H), 4.29–3.77(m, 4H), 2.59–2.50 (m, 1H), 2.40–2.30 (m, 1H), 2.10–1.52 (m, 3H), 1.48–1.45 (m, 3H), 1.43–1.30 (m, 2H), 1.16–0.97 (m, 1H), 0.88–0.81 (m, 9H), 0.00–−0.05 (m, 6H). ^{13}C-NMR (101 MHz, $CDCl_3$) δ 178.7, 178.4, 174.7, 174.1, 173.94, 173.88, 173.6, 138.6, 137.9, 137.2, 136.7, 124.7, 123.8, 122.2, 121.8, 105.6, 104.9, 104.3, 84.9, 84.8, 83.84, 83.81, 76.1, 73.6, 67.3, 66.7, 65.5, 65.2, 63.0, 62.9, 60.2, 60.1, 41.5, 40.2, 39.9, 39.6, 37.2, 37.0, 36.6, 36.3, 36.1, 32.0, 30.1, 29.8, 29.79, 29.75, 29.5, 25.93, 25.91, 25.88, 22.8, 22.2, 22.0, 21.9, 21.7, 18.9, 18.29, 18.27, 18.26, 18.18, 17.7,−4.3, −4.65, −4.69, −4.80, −4.87, −4.90, −4.91. The NMR peaks of all isomers are reported. HRMS (ESI+) calculated for $C_{20}H_{34}O_5SiNa$ [M + Na]$^+$ 405.2073, found 405.2070.

(1*E*,7*E*)-2,6-Dihydroxy-13-methoxy-10-methyl-11-oxabicyclo[8.2.1]trideca-1(13),7-dien-12-one (22):

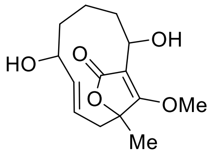

To a solution of **20** (143 mg, 0.374 mmol) in THF (3.7 mL), TBAF (1 M in THF, 3.7 mL, 3.7 mmol, 10.0 equiv) was added. The reaction mixture was stirred at room temperature for 16 h. Then, an aqueous solution of HCl (0.1 M, 4 mL) was added, followed by addition of water (10 mL) and CH_2Cl_2 (20 mL). The reaction mixture was extracted with CH_2Cl_2 (4 × 5 mL), the combined organic layers were dried over Na_2SO_4, filtered and concentrated in vacuo. Purification by flash column chromatography (CH_2Cl_2:MeOH 97:3) afforded **22** as a colorless sticky solid (73 mg, 0.272 mmol, 73%). ^{1}H-NMR (400 MHz, $CDCl_3$) δ 5.59–5.23 (m, 2H), 4.73–4.51 (m, 1H), 4.31–3.84 (m, 4H), 3.01–2.33 (m, 2H), 2.20–1.51 (m, 5H), 1.50–1.45 (m, 3H), 1.44–0.97 (m, 3H). ^{13}C-NMR (101 MHz, $CDCl_3$) δ 178.7, 178.6, 174.6, 174.4, 173.9, 173.8, 137.41, 137.35, 136.9, 136.7, 126.7, 125.9, 122.5, 122.0, 105.8, 105.7, 104.6, 104.5, 84.9, 84.7, 83.9, 83.8, 77.5, 75.6, 73.0, 67.0, 66.8, 65.3, 63.1, 63.0, 60.3, 60.2, 41.5, 40.0, 39.9, 39.5, 39.1, 37.7, 36.4, 36.0, 35.2, 34.7, 25.3, 22.2, 22.14, 22.07, 21.7, 20.3, 19.2, 17.7, 17.5. The NMR peaks of all isomers are reported. HRMS (ESI+) calculated for $C_{14}H_{20}O_5Na$ [M + Na]$^+$ 291.1208, found 291.1202.

(1*E*,7*E*)-13-Methoxy-10-methyl-11-oxabicyclo[8.2.1]trideca-1(13),7-diene-2,6,12-trione (1):

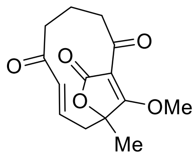

To a solution of **20** (23.0 mg, 0.086 mmol) in CH_2Cl_2 (0.4 mL), Dess–Martin periodinane (90.8 mg, 0.214 mmol, 2.5 equiv) was added. The resulting suspension was stirred at room temperature for 2 h. The reaction was quenched with an aqueous solution of $Na_2S_2O_3 \cdot 5H_2O$ (372 mg in 4 mL water), and the mixture was stirred for 10 min (until the suspension becomes a transparent solution). CH_2Cl_2 (5 mL) and water (5 mL) were added, and the layers were separated. The aqueous layer was extracted with CH_2Cl_2 (3 × 5 mL), and the combined organic layers were washed with a saturated aqueous solution of $NaHCO_3$ (1 × 10 mL), brine (1 × 10 mL), dried over Na_2SO_4, filtered and concentrated in vacuo. Purification by flash column chromatography (hexane:EtOAc 7:3) afforded **1** as a white sticky solid (5.5 mg, 0.0208 mmol, 24%). ^{1}H-NMR (400 MHz, $CDCl_3$) δ 6.34 (ddd, J = 16.4, 9.0, 6.5 Hz, 1H), 6.15 (dt, J = 16.4, 0.7 Hz, 1H), 4.19 (s, 3H), 2.92 (ddd, J = 13.8, 6.4, 4.8 Hz, 1H), 2.80 (ddd, J = 12.9, 6.5, 0.7 Hz, 1H), 2.56–2.47 (m, 4H), 2.22–2.10 (m, 1H), 1.93–1.83 (m, 1H), 1.60 (s, 3H). ^{13}C-NMR (101 MHz, $CDCl_3$) δ 201.3, 198.3, 177.9, 169.3, 137.5, 135.1, 107.9, 84.8, 63.0, 41.4, 40.4, 40.3, 21.7, 21.2. HRMS (ESI+) calculated for $C_{14}H_{16}O_5Na$ [M + Na]$^+$ 287.0895, found 287.0888.

5-Allyl-3-(5-((*tert*-Butyldimethylsilyl)oxy)-1-hydroxyhept-6-en-1-yl)-4-methoxy-5-phenethylfuran-2(5*H*)-one (19):

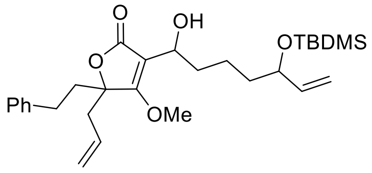

To a solution of iPr_2NH (filtered over a short column of basic Al_2O_3 just before the reaction, 0.50 mL, 3.53 mmol, 1.5 equiv) in anhydrous THF (5 mL) at –78 °C under a nitrogen atmosphere, *n*-BuLi (2.5 M in hexanes, 1.4 mL, 3.53 mmol, 1.5 equiv) was added. The reaction mixture was stirred at –78 °C for 30 min. A solution of **5** (606 mg, 2.35 mmol, 1.0 equiv) in anhydrous THF (1 mL) was added and the reaction mixture was stirred at –78 °C for 30 min. Then, a solution of **3** (625 mg, 2.58 mmol, 1.1 equiv) in anhydrous THF (2 mL) was added and the reaction mixture was stirred at –78 °C for 1.5 h, and then it was allowed to warm up to room temperature. The reaction was quenched with water (15 mL) and extracted with EtOAc (3 × 10 mL). The combined organic layers were dried over Na_2SO_4, filtered and concentrated in vacuo. Purification by flash column chromatography (hexane:EtOAc 85:15) afforded **19** as a pale yellow oil (697 mg, 1.39 mmol, 59%). ^{1}H-NMR (400 MHz, $CDCl_3$) δ 7.30–7.25 (m, 2H, overlaps with $CDCl_3$ signal), 7.21–7.12 (m, 3H), 5.82–5.56 (m, 2H), 5.16–4.94 (m, 4H), 4.74–4.67 (m, 1H), 4.11–4.05 (m, 1H), 4.03 and 3.93–3.90 (m, 3H), 2.68–2.37 (m, 4H), 2.17–1.97 (m, 2H), 1.95–1.84 (m, 1H), 1.73–1.65 (m, 1H),1.59–1.29 (m, 4H), 0.89–0.86 (m, 9H), 0.05–0.00 (m, 6H). ^{13}C-NMR (101 MHz, $CDCl_3$) δ 173.34, 173.33, 173.27, 173.26, 173.22, 173.19, 172.7, 141.68, 141.65, 141.62, 141.56, 140.9, 140.8, 130.50, 130.49, 130.46, 128.60, 128.59, 128.54, 128.48, 128.43, 126.3, 126.2, 126.1, 120.10, 120.08, 120.07, 120.06, 119.6, 114.0, 105.65, 105.63, 85.26, 85.22, 85.21, 74.0, 73.9, 73.73, 73.68, 66.7, 66.62, 66.59, 66.49, 60.3, 59.90, 59.89, 41.0, 40.90, 40.89, 38.26, 38.25, 38.24, 37.96, 37.91, 37.84, 37.39, 37.32, 37.30, 29.30, 29.28, 29.21, 26.01, 25.99, 22.1, 22.0, 21.9, 21.8, 18.38, 18.35, –4.21, –4.24, –4.25, –4.27, –4.67, –4.69, –4.70. The NMR peaks of all isomers are reported. HRMS (ESI+) calculated for $C_{29}H_{44}O_5SiNa$ [M + Na]$^+$ 523.2856, found 523.2852.

(1*E*,7*E*)-6-((*tert*-Butyldimethylsilyl)oxy)-2-hydroxy-13-methoxy-10-phenethyl-11-oxabicyclo[8.2.1]trideca-1(13),7-dien-12-one (21):

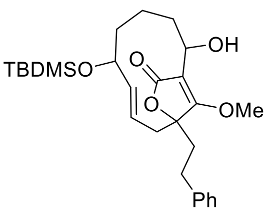

To a solution of **19** (300 mg, 0.600 mmol) in 1,2-dichloroethane (290 mL) at 103 °C (pre-heated oil bath), a solution of Hoveyda–Grubbs second generation catalyst (19.0 mg, 30 µmol, 5 mol %) in 1,2-dichloroethane (10 mL) was added (the final concentration of substrate was 0.002 M). The reaction mixture was stirred at reflux for 1 h. Then, the reaction was cooled down and the solvent evaporated in vacuo. Purification by flash column chromatography (hexane: EtOAc 9:1) afforded **19** as a yellow oil (129 mg, 0.272 mmol, 45%). ^{1}H-NMR (400 MHz, $CDCl_3$) δ 7.30–7.25 (m, 2H, overlaps with $CDCl_3$ signal), 7.21–7.14 (m, 3H), 5.57–5.21 (m, 2H), 4.66–4.50 (m, 1H), 4.31–3.80 (m, 4H), 2.75–2.58 (m, 2H), 2.53–2.31 (m, 2H), 2.19–2.05 (m, 2H), 1.92–1.26 (m, 5H), 1.15–1.07 (m, 1H), 0.91–0.83 (m, 9H), 0.03–−0.03 (m, 6H). ^{13}C-NMR (101 MHz, $CDCl_3$) δ 177.4, 174.3, 174.1, 173.4, 172.7, 141.1, 141.0, 140.8, 138.7, 138.1, 128.64, 128.60, 128.57, 128.53, 128.50, 126.3, 126.23, 126.21, 124.3, 123.5, 121.4, 106.9, 105.9, 87.2, 86.1, 76.2, 73.7, 67.4, 66.9, 65.3, 62.9, 59.93, 59.89, 41.3, 39.6, 39.2, 38.9, 37.1, 36.72, 36.66, 36.55, 36.44, 36.17, 32.1, 29.82, 29.66, 29.62, 29.55, 26.02, 25.95, 25.93, 25.90, 22.8, 19.0, 18.32, 18.28, 18.21, 17.7,–4.29, –4.31, –4.61, –4.67, –4.84, –4.90. The NMR peaks of all isomers are reported. HRMS (ESI+) calculated for $C_{27}H_{40}O_5SiNa$ $[M + Na]^+$ 495.2543, found 495.2541.

(1*E*,7*E*)-2,6-Dihydroxy-13-methoxy-10-phenethyl-11-oxabicyclo[8.2.1]trideca-1(13),7-dien-12-one (23):

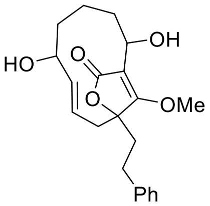

To a solution of **21** (120 mg, 0.254 mmol) in THF (2.5 mL), TBAF (1 M in THF, 2.5 mL, 2.5 mmol, 10.0 equiv) was added. The reaction mixture was stirred at room temperature for 16 h. Then, an aqueous solution of HCl (0.1 M, 4 mL) was added, followed by addition of water (10 mL) and CH_2Cl_2 (20 mL). The reaction mixture was extracted with CH_2Cl_2 (4 × 5 mL), the combined organic layers were dried over Na_2SO_4, filtered and concentrated in vacuo. Purification by flash column chromatography (CH_2Cl_2:MeOH 98:2) afforded **23** as a colorless sticky solid (68 mg, 0.189 mmol, 74%). ^{1}H-NMR (400 MHz, $CDCl_3$) δ 7.30–7.25 (m, 2H, overlaps with $CDCl_3$ signal), 7.21–7.15 (m, 3H), 5.57–5.25 (m, 2H), 4.70–4.51 (m, 1H), 4.35–3.83 (m, 4H), 2.76–2.33 (m, 4H), 2.21–2.05 (m, 3H), 2.02–0.97 (m, 7H). ^{13}C-NMR (101 MHz, $CDCl_3$) δ 177.3, 174.2, 174.1, 173.4, 173.0, 141.0, 140.8, 140.7, 137.6, 137.4, 137.1, 128.56, 128.54, 128.51, 128.49, 128.48, 128.47, 126.29, 126.24, 126.19, 126.13, 125.4, 107.1, 106.00, 105.97, 87.1, 86.9, 86.1, 75.5, 73.0, 67.0, 66.7, 65.1, 65.1, 63.0, 62.8, 60.0, 59.9, 41.2, 39.3, 39.1, 38.4, 37.7, 36.8, 36.5, 36.4, 36.3, 35.9, 34.6, 29.57, 29.51, 29.48, 20.1, 19.2, 17.7. The NMR peaks of all isomers are reported. HRMS (ESI+) calculated for $C_{21}H_{26}O_5Na$ $[M + Na]^+$ 381.1678, found 381.1675.

(1*E*,7*E*)-13-Methoxy-10-phenethyl-11-oxabicyclo[8.2.1]trideca-1(13),7-diene-2,6,12-trione (2):

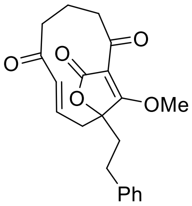

To a solution of **23** (50.0 mg, 0.139 mmol) in CH_2Cl_2 (0.6 mL), Dess–Martin periodinane (148 mg, 0.349 mmol, 2.5 equiv) was added. The resulting suspension was stirred at room temperature for 2 h. The reaction was quenched with an aqueous solution of $Na_2S_2O_3\cdot5H_2O$ (606 mg in 5 mL water), and the mixture was stirred for 10 min (until the suspension becomes a transparent solution). CH_2Cl_2 (5 mL) and water (5 mL) were added, and the layers were separated. The aqueous layer was extracted with CH_2Cl_2 (3 × 5 mL), and the combined

organic layers were washed with a saturated aqueous solution of $NaHCO_3$ (1 × 10 mL), brine (1 × 10 mL), dried over Na_2SO_4, filtered and concentrated in vacuo. Purification by flash column chromatography (hexane:EtOAc 7:3) afforded **2** as a white sticky solid (20.1mg, 0.0567 mmol, 41%). ^{1}H-NMR (400 MHz, $CDCl_3$) δ 7.32–7.26 (m, 2H, overlaps with $CDCl_3$ signal), 7.23–7.14 (m, 3H), 6.34 (ddd, J = 15.9, 8.9, 6.7 Hz, 1H), 6.17–6.13 (m, 1H), 4.04 (s, 3H), 2.95–2.73 (m, 3H), 2.59–2.45 (m, 5H), 2.27–2.21 (m, 2H), 2.19–2.10 (m, 1H), 1.93–1.83 (m, 1H). ^{13}C-NMR (101 MHz, $CDCl_3$) δ 201.3, 197.8, 177.3, 169.6, 140.2, 137.3, 135.3, 128.7, 128.5, 126.6, 109.1, 87.0, 63.0, 41.2, 40.1, 39.6, 36.3, 29.6, 21.9. HRMS (ESI+) calculated for $C_{21}H_{22}O_5Na$ $[M + Na]^+$ 377.1365, found 377.1359.

1.3 Biological evaluation: antibacterial activity

Test of the resistant pattern of different strains were made in a 96-well-plate-based broth screening. Test plates were freshly prepared by to each well add 80 µL cation-adjusted Mueller-Hinton broth (CA-MHB) (Oxoid) supplemented with the substrate (compounds **1**, **2**, **4** and **5**) to a final concentration of either 200, 150, 100, 50, 25, 12.5, 6.25 or 3.125 g/mL. As the substrate (compounds **1**, **2**, **4** and **5**) was dissolved in DMSO all wells had a final concentration of 5% DMSO. All concentrations were done in triplicates for each strain. As a negative control the strains were cultured in CA-MHB with 5% DMSO and as a positive control the strains were grown in CA-MHB with 5% DMSO and vancomycin. Inoculum was prepared as followed: bacteria were dissolved in 0.85% NaCl to an OD of 0.14 and then diluted 40 times in CA-MHB. 20 µL of the suspension were then added to the respective well in the test-plates. The following strains were tested: *Staphylococcus aureus* (CCUG38266), *S. aureus* (CCUG58065) and *S. aureus* (ATCC28213/CCUG15915). Plates were incubated at 37 °C, 180 rpm for 18 h and results read by the unaided eye.

2. References

(1) Parsons, J. F.; Jensen, P. Y.; Pachikara, A. S.; Howard, A. J.; Eisenstein, E.; Ladner, J. E. *Biochemistry* **2002**, *41*, 2198–2208.

(2) Pronk, S.; Páll, S.; Schulz, R.; Larsson, P.; Bjelkmar, P.; Apostolov, R.; Shirts, M. R.; Smith, J. C.; Kasson, P. M.; Van Der Spoel, D.; Hess, B; Lindahl, E. *Bioinformatics* **2013**, *29*, 845–854.

(3) Abraham, M. J.; Murtola, T.; Schulz, R.; Páll, S.; Smith, J. C.; Hess, B.; Lindah, E. *SoftwareX* **2015**, *1–2*, 19–25.

(4) Schrödinger Release 2017-2: Schrödinger, LLC, New York, NY, 2017.

(5) Friesner, R. A.; Murphy, R. B.; Repasky, M. P.; Frye, L. L.; Greenwood, J. R.; Halgren, T. A.; Sanschagrin, P. C.; Mainz, D. T. *J. Med. Chem.* **2006**, *49*, 6177–6196.

(6) Zhu, K.; Borrelli, K. W.; Greenwood, J.; Day, T.; Abel, R.; Farid, R.; Harder, E. *J. Chem. Inf. Model.* **2014**, *54*, 1932–1940.

(7) Frankowski, K. J.; Golden, J. E.; Zeng, Y.; Lei, Y.; Aubé, J. *J. Am. Chem. Soc.* **2008**, *130*, 6018–6024.

(8) Shelke, A. M.; Suryavanshi, G. *Tetrahedron Lett.* **2015**, *56* (45), 6207–6209.

(9) Shelke, A. M.; Rawat, V.; Suryavanshi, G.; Sudalai, A. *Tetrahedron Asymmetry* **2012**, *23*, 1534–1541.

(10) Reilly, M. K.; Rychnovsky, S. D. *Org. Lett.* **2010**, *12*, 4892–4895.

(11) Kim, S. H.; Kim, K. H.; Kim, J. N. *Adv. Synth. Catal.* **2011**, *353*, 3335–3339.

3. NMR spectra

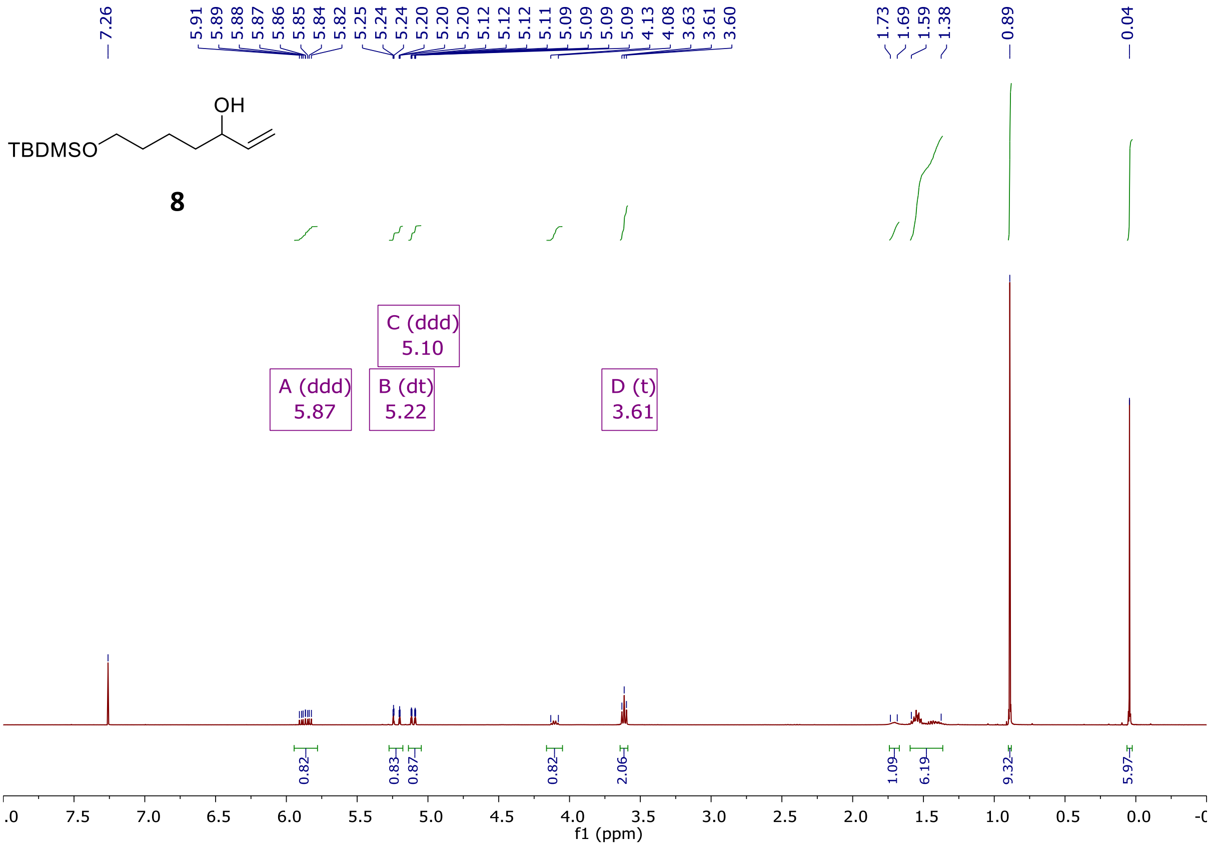

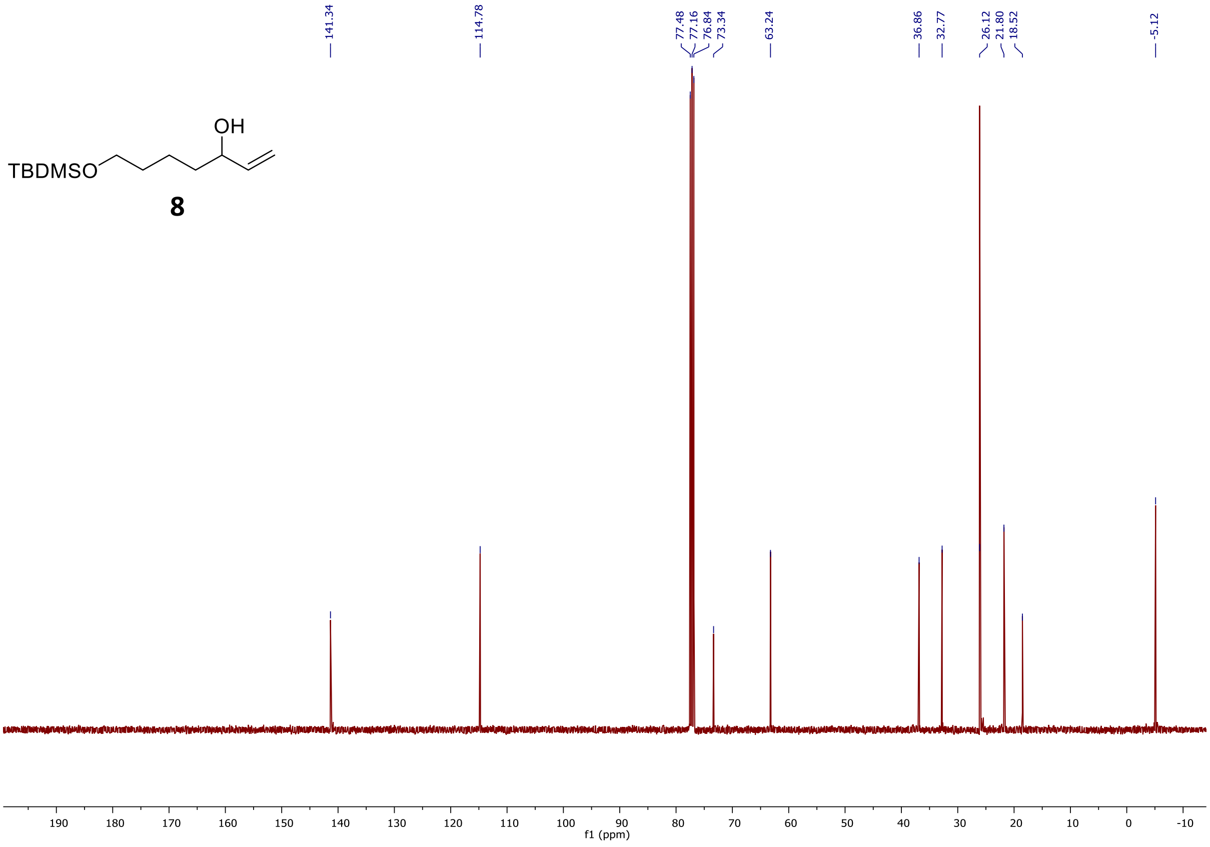

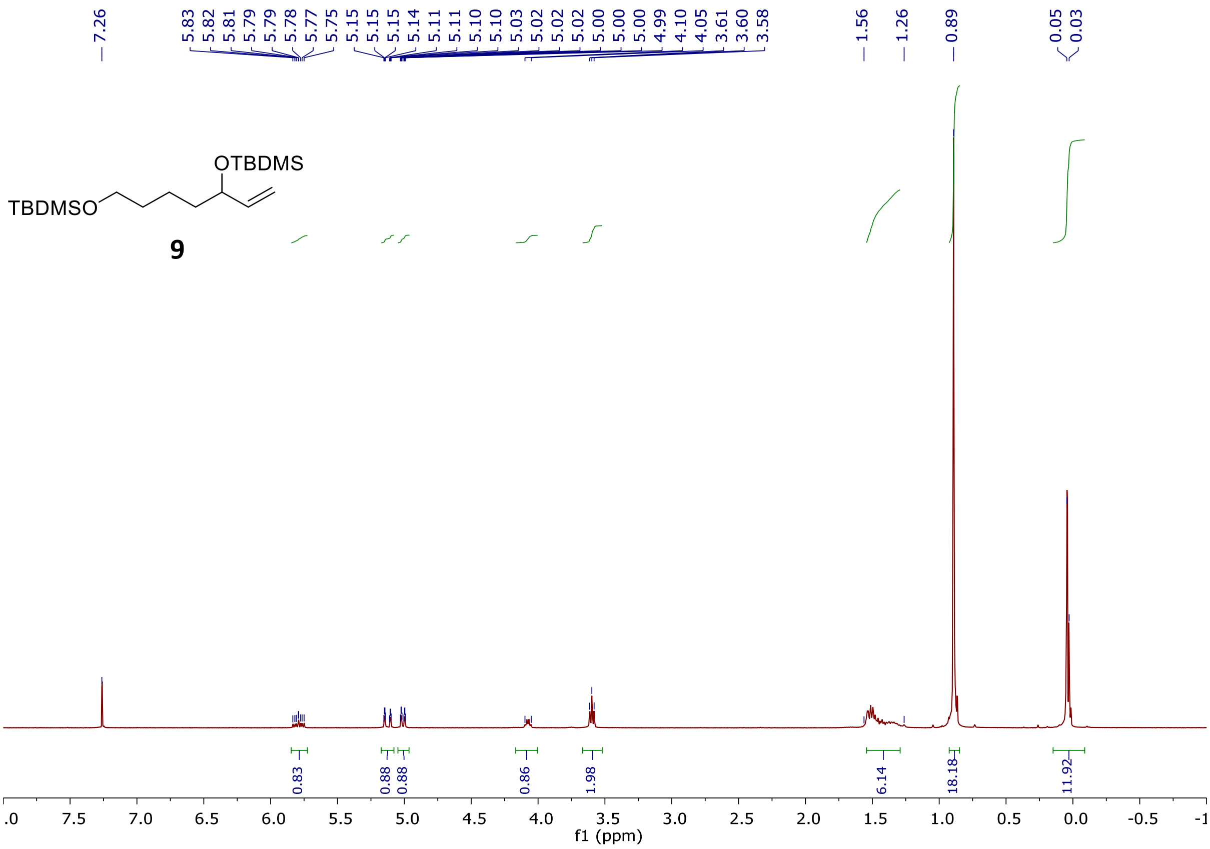
7.26
5.83
5.82
5.81
5.79
5.78
5.77
5.75
5.15
5.15
5.15
5.14
5.11
5.11
5.10
5.10
5.03
5.02
5.02
5.02
5.00
5.00
5.00
4.99
4.10
4.05
3.61
3.60
3.58
1.56
1.26
0.89
0.05
0.03
OTBDMS
TBDMSO
9
0.83
0.88
0.88
0.86
1.98
6.14
18.18
11.92
f1 (ppm)

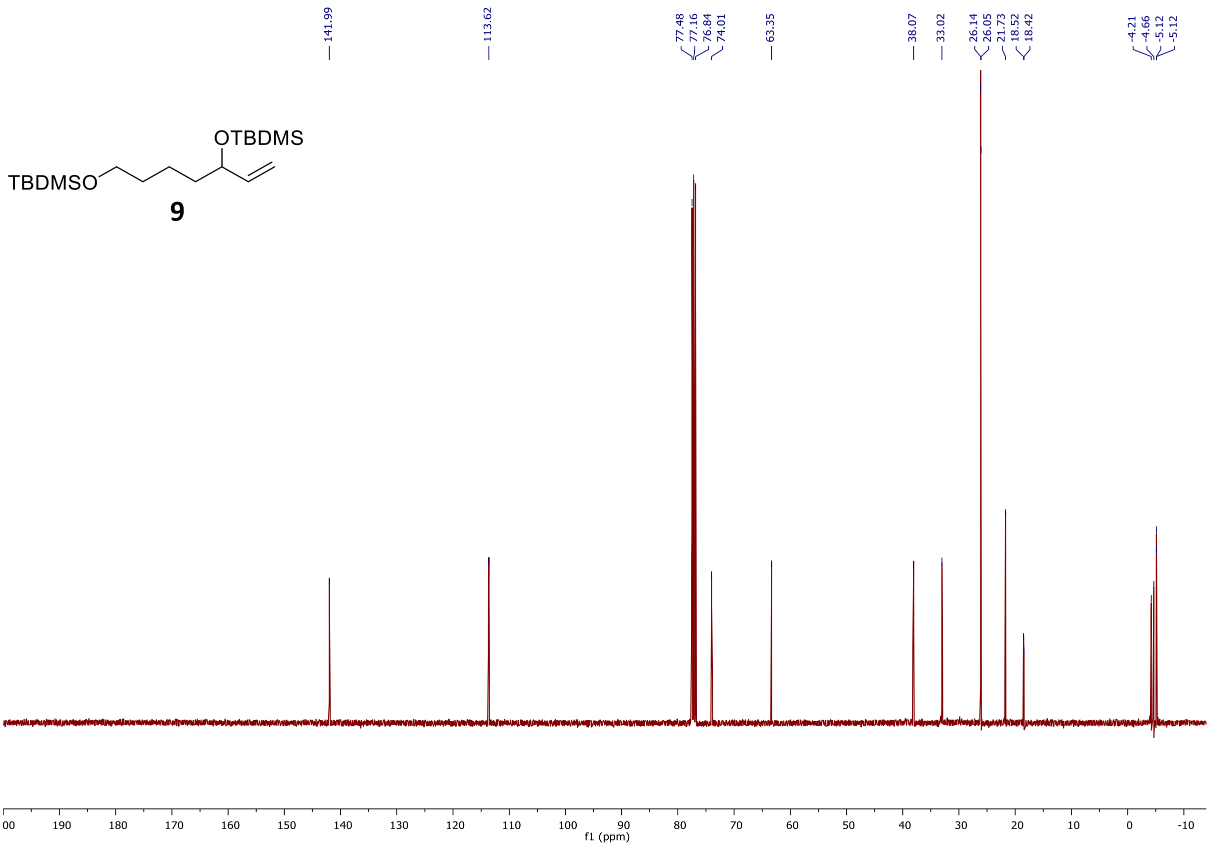
141.99
113.62
77.48
77.16
76.84
74.01
63.35
38.07
33.02
26.14
26.05
21.73
18.52
18.42
-4.21
-4.66
-5.12
-5.12
OTBDMS
TBDMSO
9
f1 (ppm)

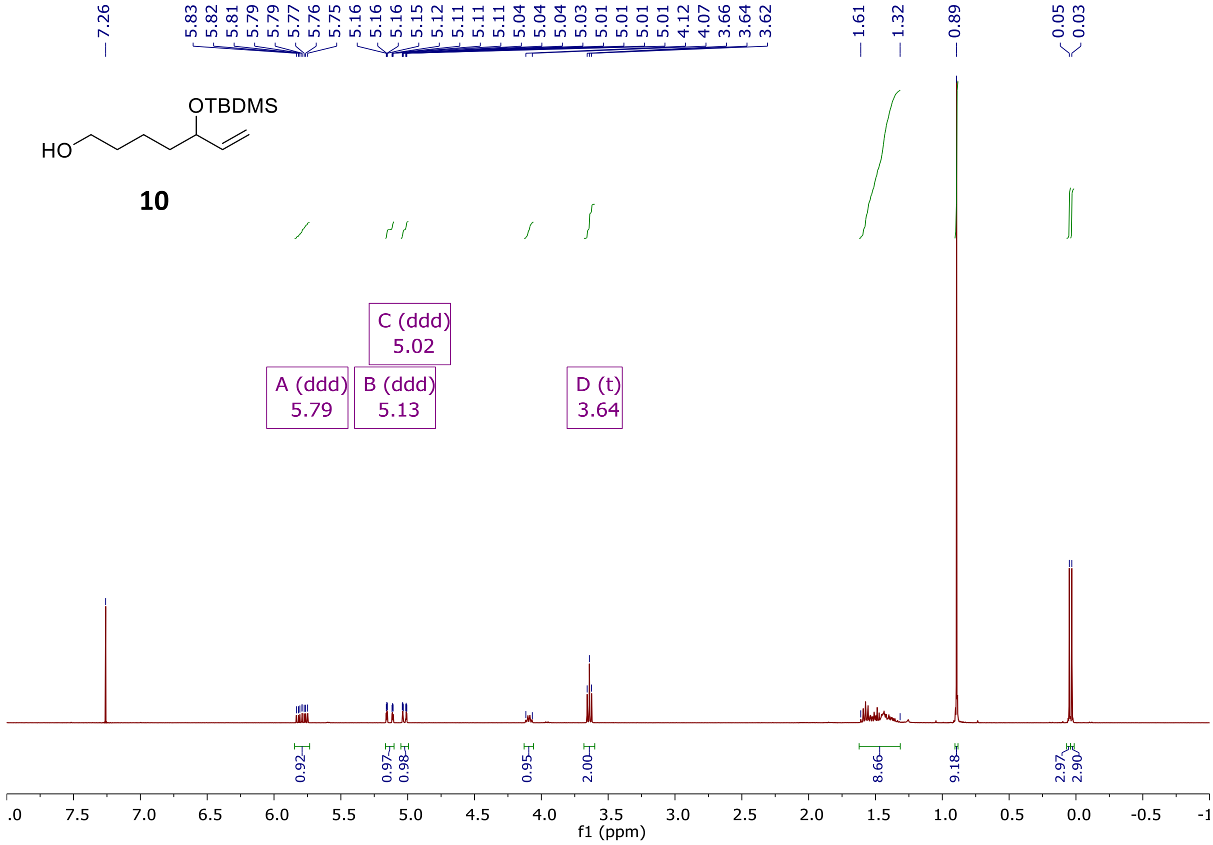
OTBDMS
HO
10
7.26
5.83
5.82
5.81
5.79
5.79
5.77
5.76
5.75
5.16
5.16
5.16
5.15
5.12
5.11
5.11
5.11
5.04
5.04
5.04
5.03
5.01
5.01
5.01
5.01
4.12
4.07
3.66
3.64
3.62
1.61
1.32
0.89
0.05
0.03
C (ddd) 5.02
A (ddd) 5.79
B (ddd) 5.13
D (t) 3.64
0.92
0.97
0.98
0.95
2.00
8.66
9.18
2.97
2.90
f1 (ppm)

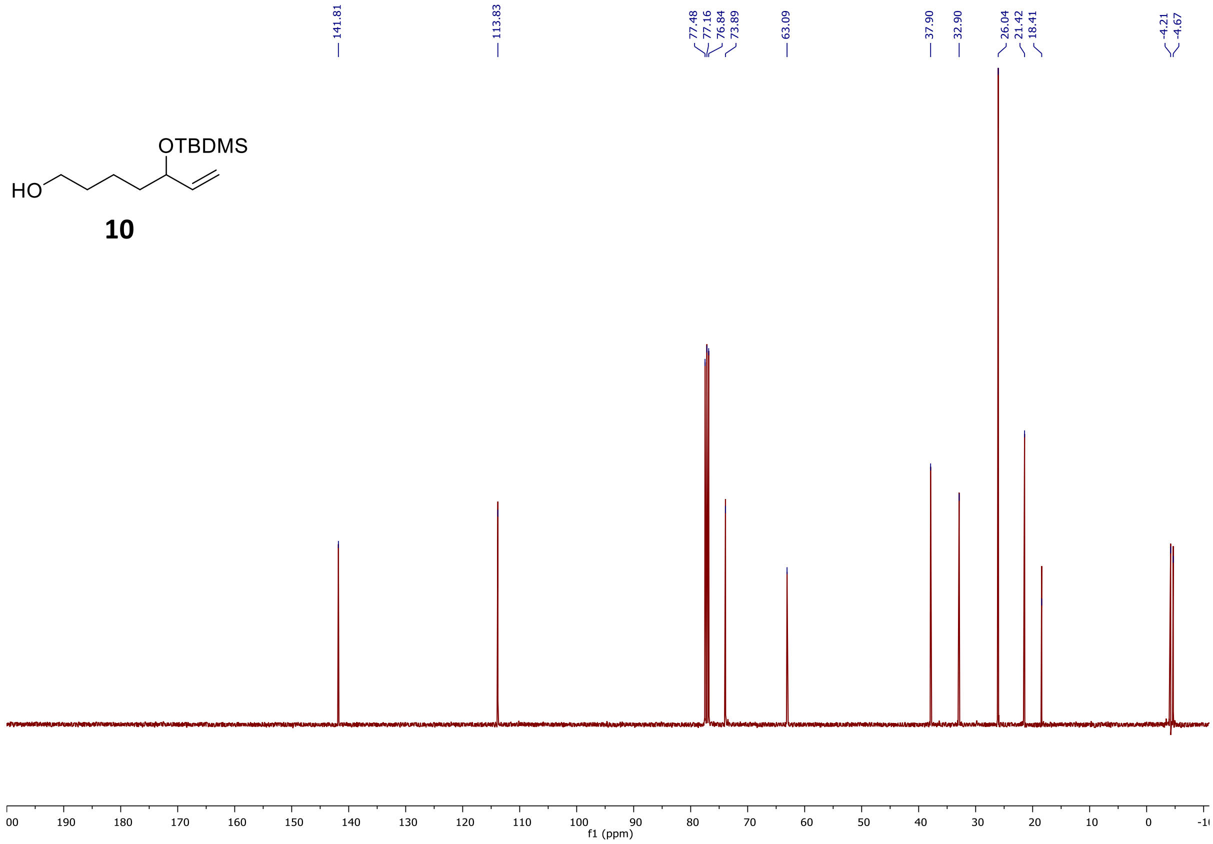
141.81
113.83
77.48
77.16
76.84
73.89
63.09
37.90
32.90
26.04
21.42
18.41
-4.21
-4.67
OTBDMS
HO
10
f1 (ppm)

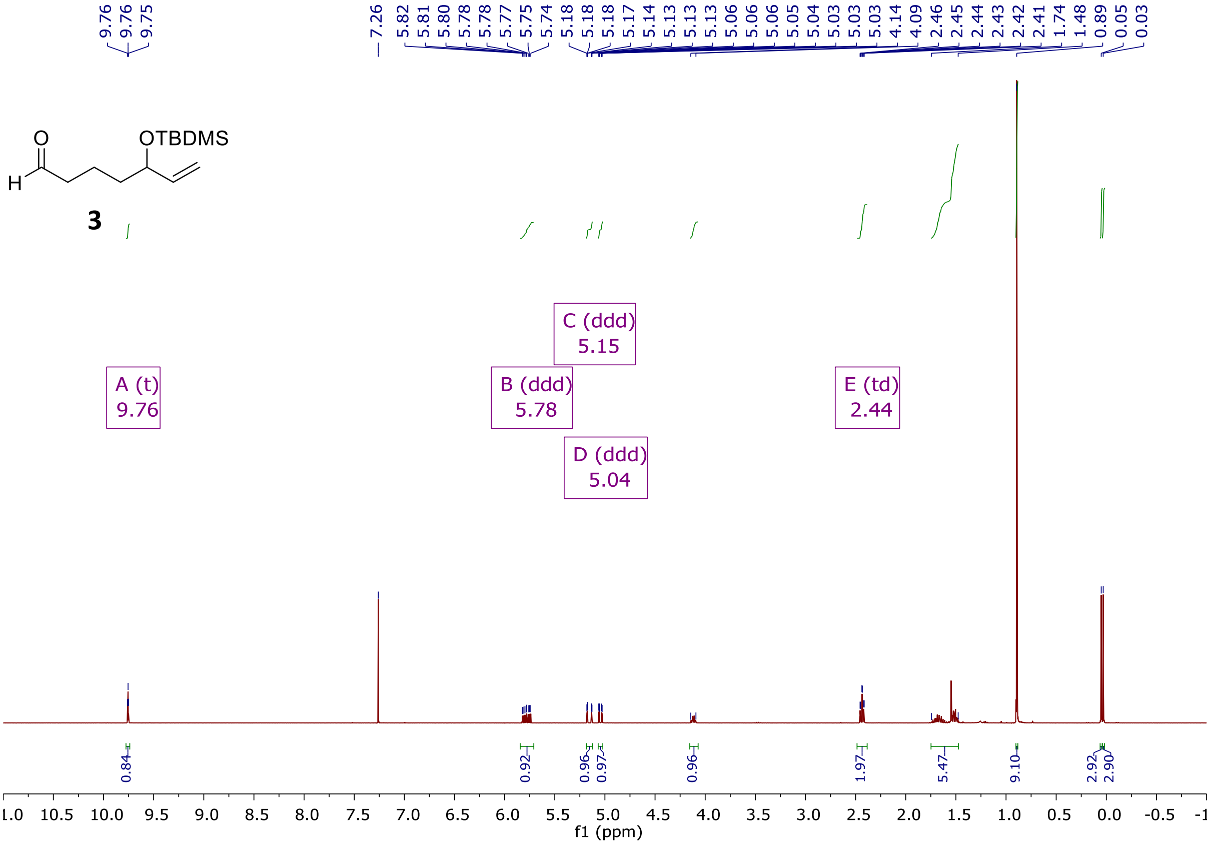
9.76 9.76 9.75
7.26
5.82 5.81 5.80 5.78 5.78 5.77 5.75 5.74 5.18 5.18 5.18 5.17 5.14 5.13 5.13 5.13 5.06 5.06 5.06 5.05 5.04 5.03 5.03 5.03 4.14 4.09 2.46 2.45 2.44 2.43 2.42 2.41 1.74 1.48 0.89 0.05 0.03
O
OTBDMS
H
3
A (t)
9.76
B (ddd)
5.78
C (ddd)
5.15
D (ddd)
5.04
E (td)
2.44
0.84
0.92
0.96
0.97
0.96
1.97
5.47
9.10
2.92
2.90
f1 (ppm)

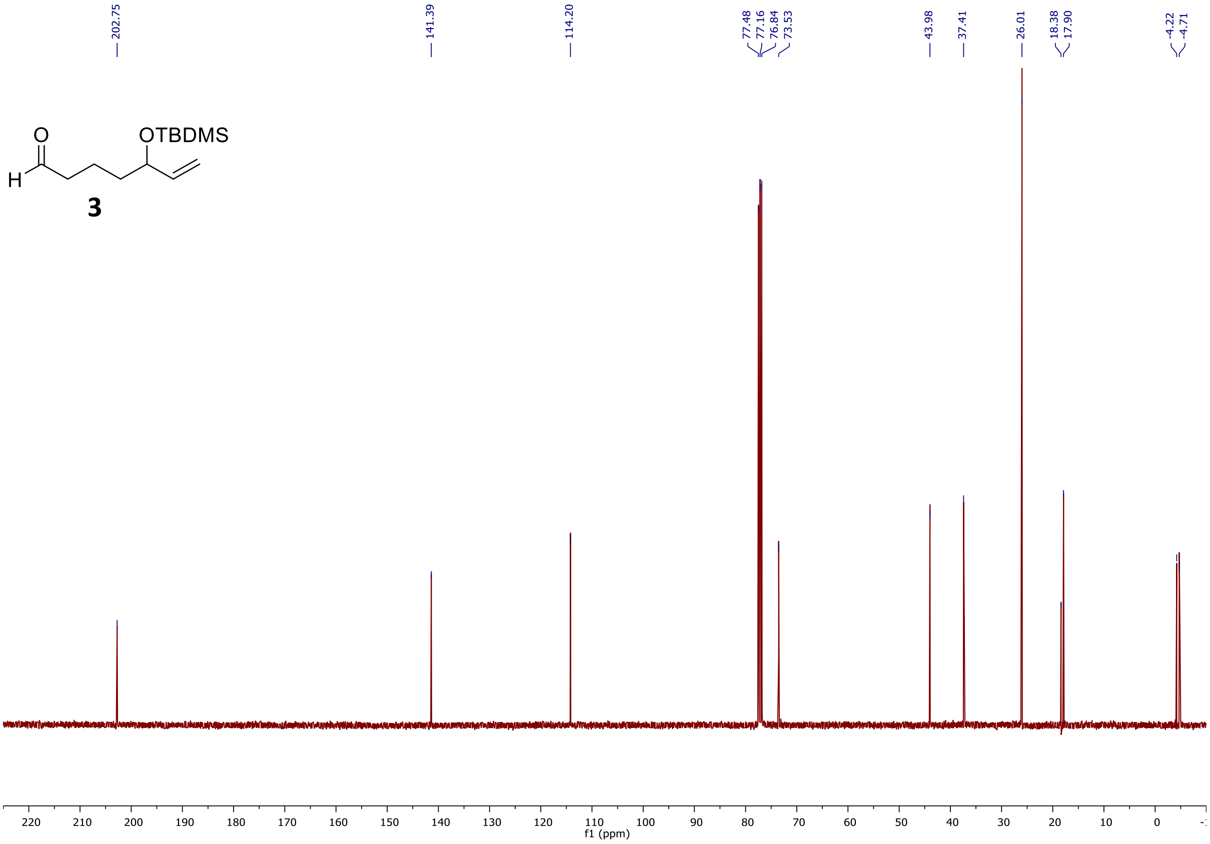
202.75
141.39
114.20
77.48 77.16 76.84 73.53
43.98
37.41
26.01
18.38 17.90
-4.22 -4.71
O
OTBDMS
H
3
f1 (ppm)

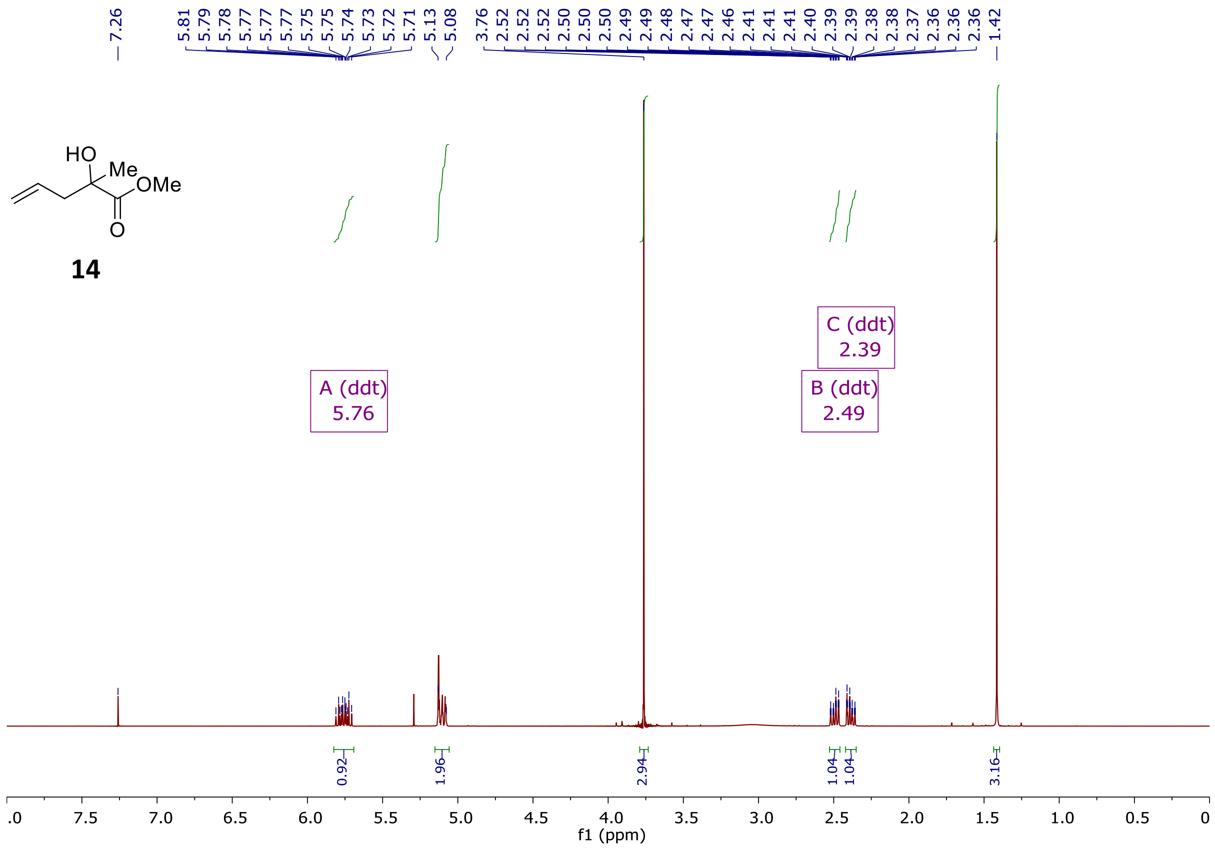
7.26
5.81 5.79 5.78 5.77 5.77 5.77 5.75 5.75 5.74 5.73 5.72 5.71 5.13 5.08
3.76 2.52 2.52 2.52 2.50 2.50 2.49 2.49 2.48 2.47 2.47 2.46 2.41 2.41 2.41 2.40 2.39 2.39 2.38 2.38 2.37 2.36 2.36 2.36 1.42
HO
Me
OMe
O
14
A (ddt)
5.76
B (ddt)
2.49
C (ddt)
2.39
0.92
1.96
2.94
1.04
1.04
3.16
f1 (ppm)

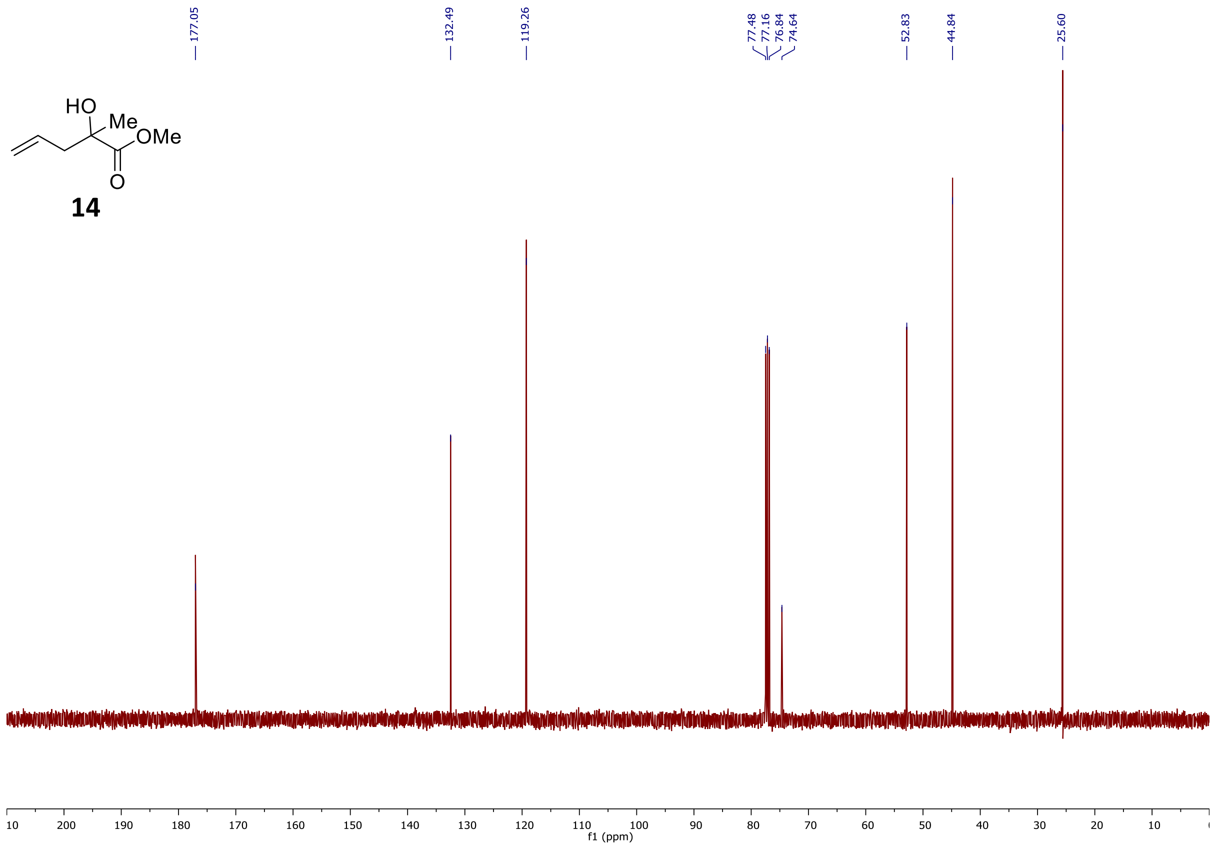
177.05
132.49
119.26
77.48
77.16
76.84
74.64
52.83
44.84
25.60
HO
Me
OMe
O
14
f1 (ppm)

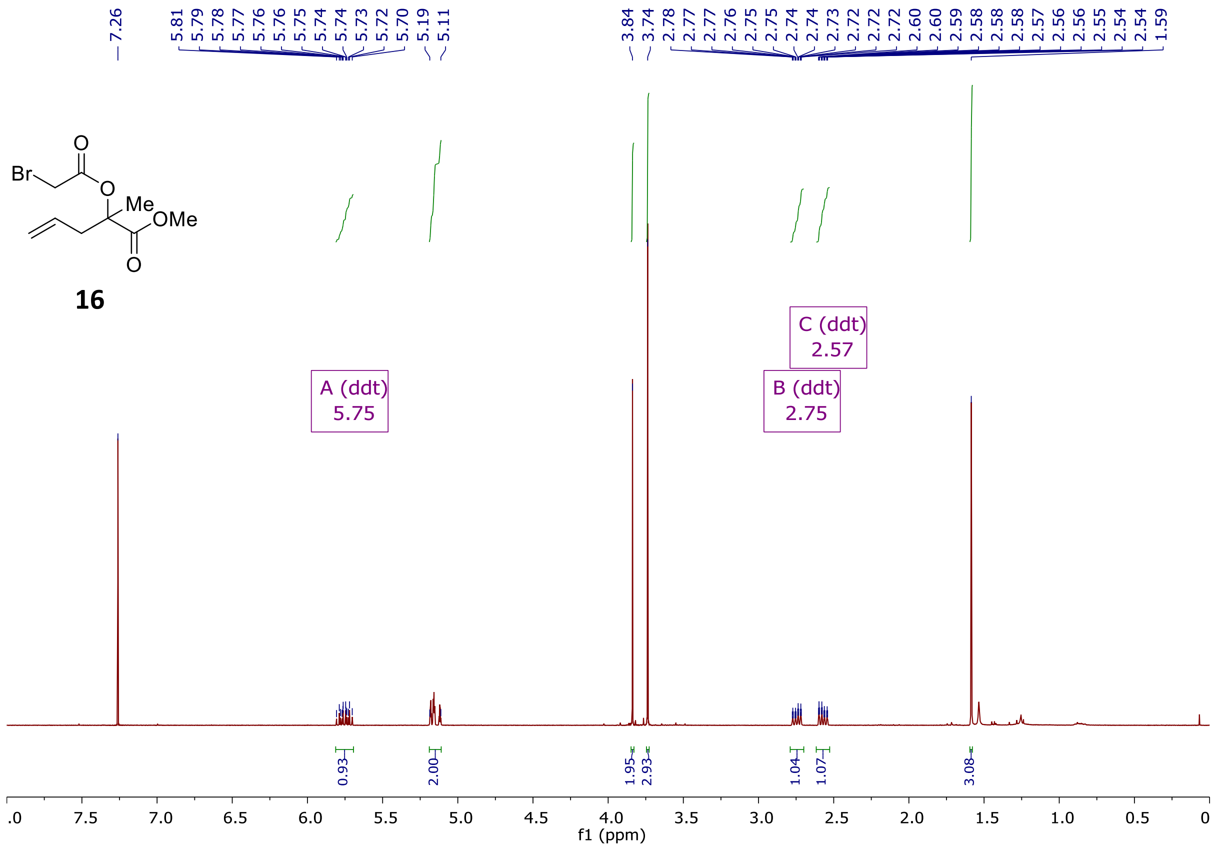
7.26
5.81 5.79 5.78 5.77 5.76 5.76 5.75 5.74 5.74 5.73 5.72 5.70 5.19 5.11
3.84 3.74 2.78 2.77 2.77 2.76 2.75 2.75 2.74 2.74 2.73 2.72 2.72 2.72 2.60 2.59 2.58 2.58 2.58 2.57 2.56 2.56 2.55 2.54 2.54 1.59
Br
O
O
Me
OMe
O
16
A (ddt)
5.75
B (ddt)
2.75
C (ddt)
2.57
0.93
2.00
1.95
2.93
1.04
1.07
3.08
f1 (ppm)

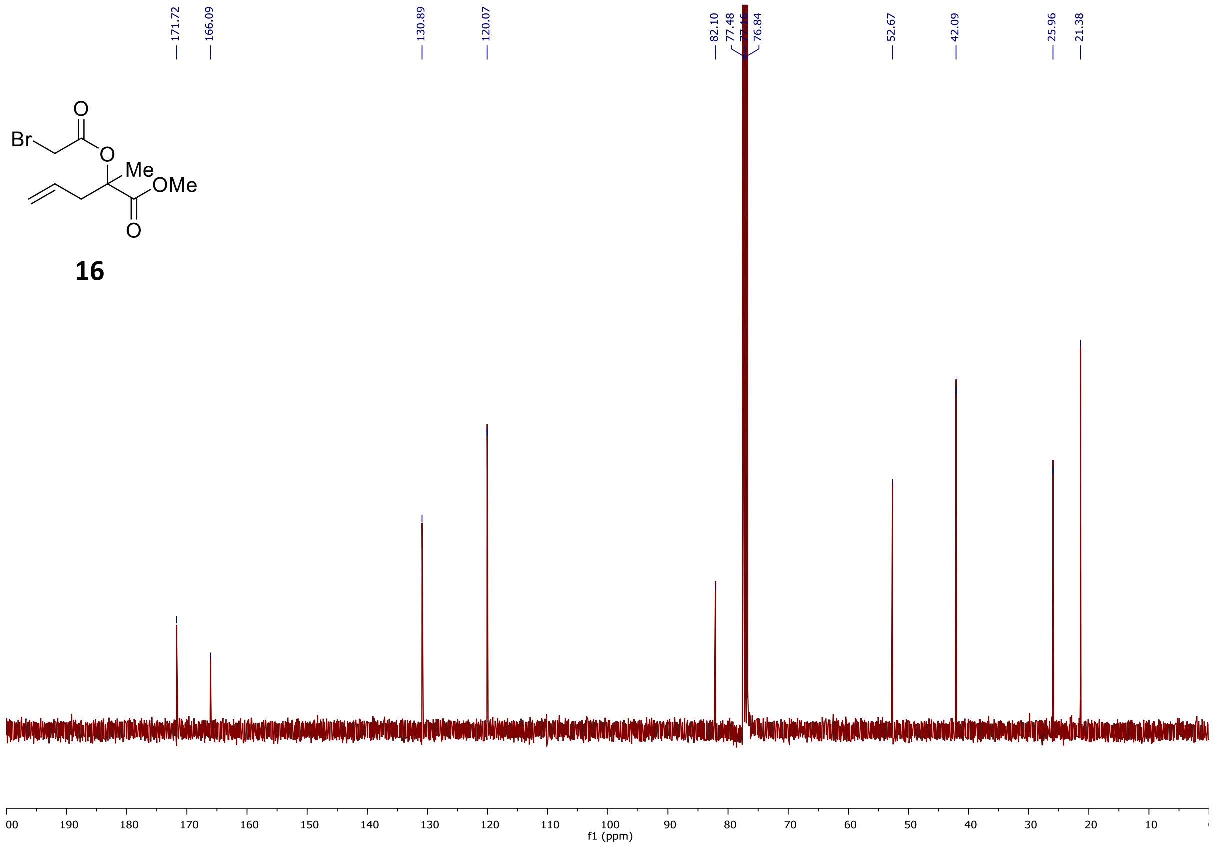
171.72
166.09
130.89
120.07
82.10
77.48
76.84
52.67
42.09
25.96
21.38
Br
O
O
Me
OMe
O
16
f1 (ppm)

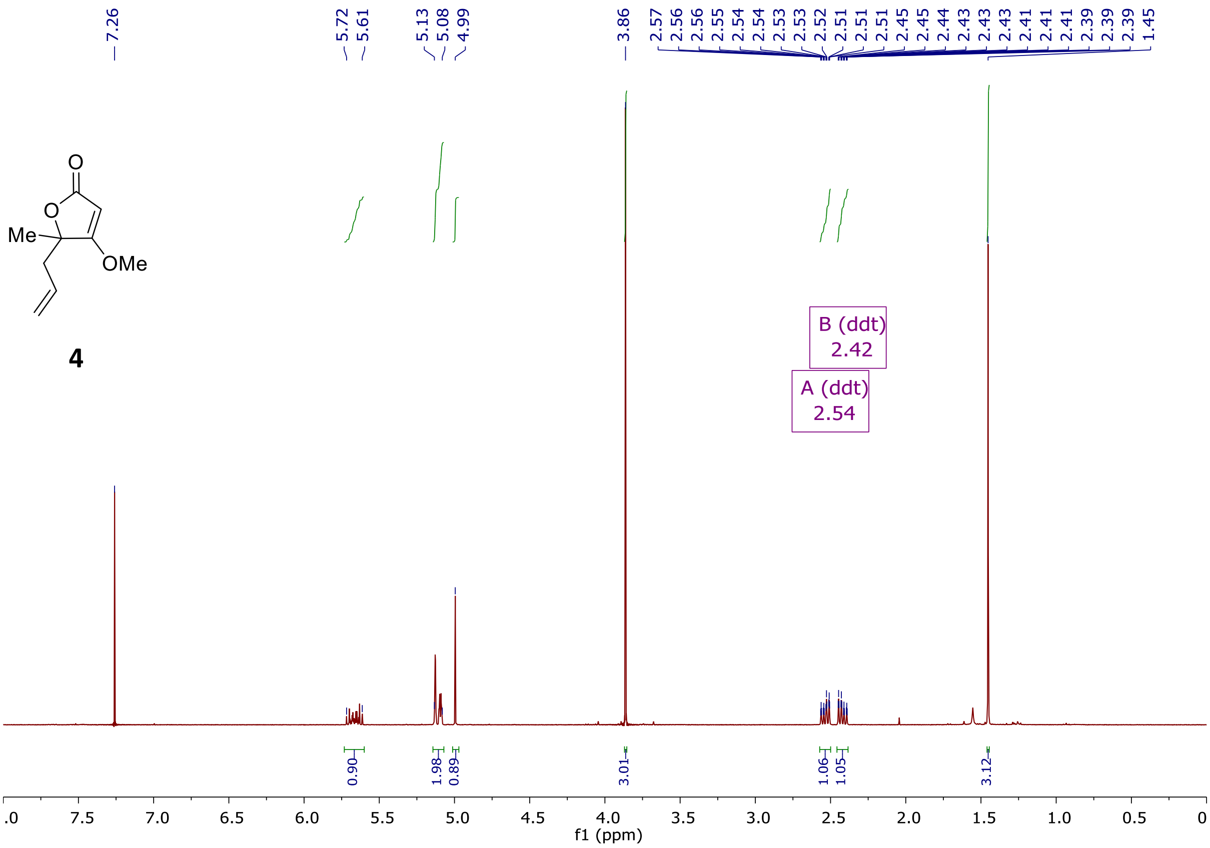
7.26
5.72
5.61
5.13
5.08
4.99
3.86
2.57 2.56 2.56 2.55 2.54 2.54 2.53 2.53 2.52 2.51 2.51 2.51 2.45 2.45 2.44 2.43 2.43 2.43 2.41 2.41 2.39 2.39 2.39
1.45
O
O
Me
OMe
4
B (ddt)
2.42
A (ddt)
2.54
0.90
1.98
0.89
3.01
1.06
1.05
3.12
f1 (ppm)

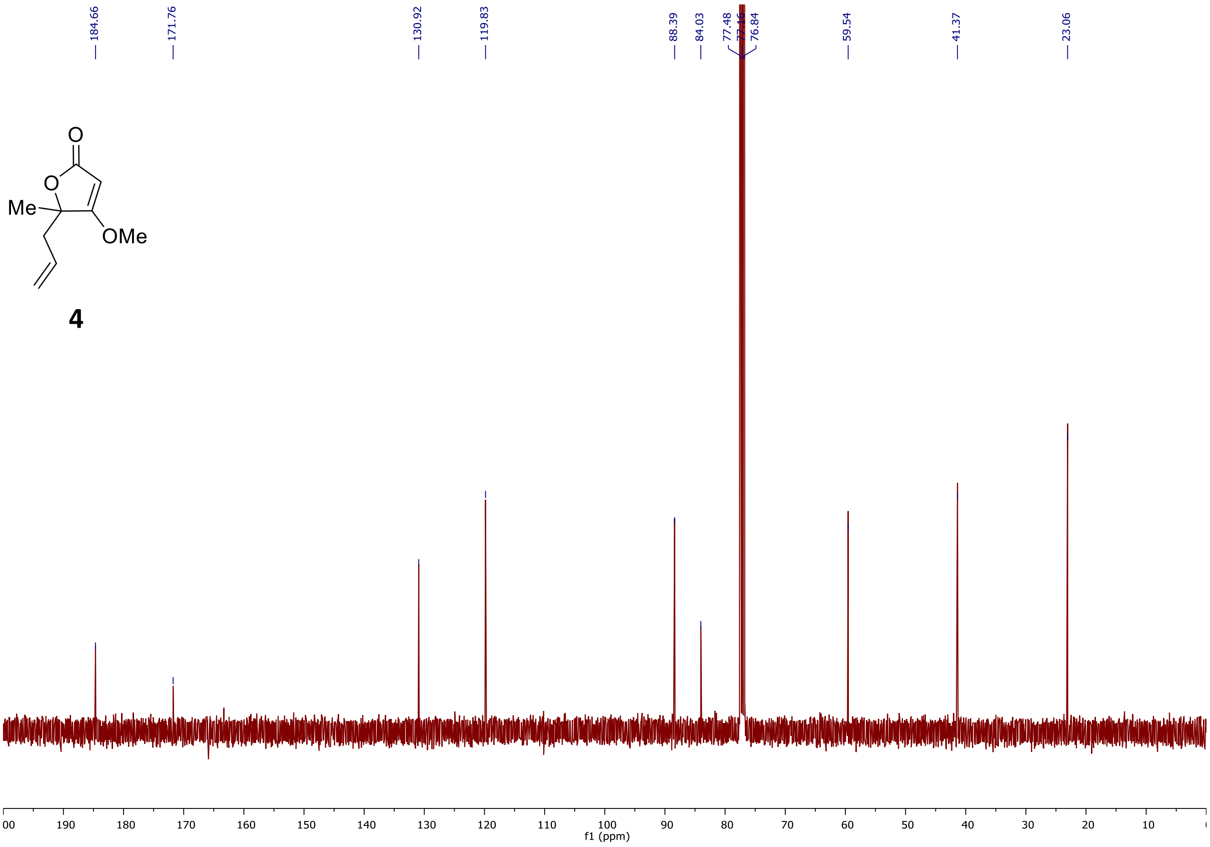
184.66
171.76
130.92
119.83
88.39
84.03
77.48
76.84
59.54
41.37
23.06
O
O
Me
OMe
4
f1 (ppm)

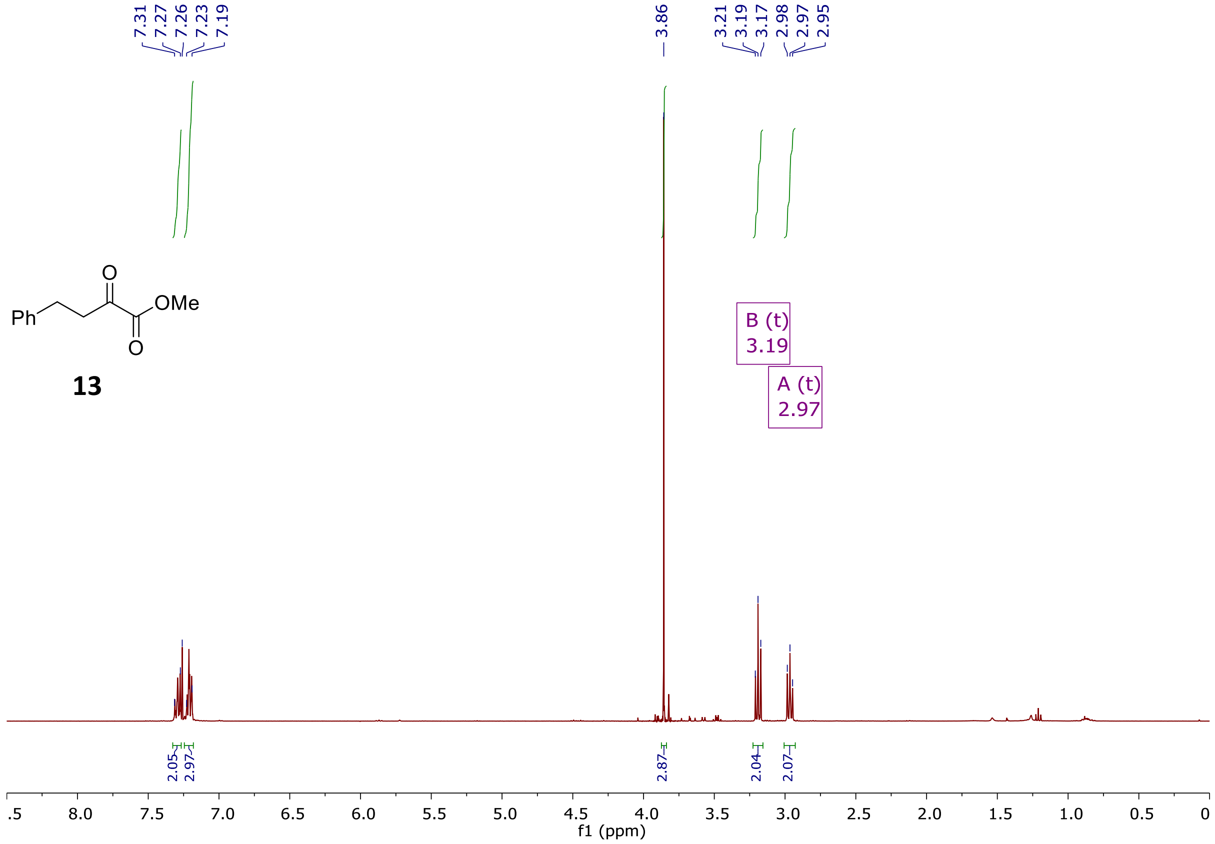
7.31
7.27
7.26
7.23
7.19
3.86
3.21
3.19
3.17
2.98
2.97
2.95
O
OMe
Ph
O
13
B (t)
3.19
A (t)
2.97
2.05
2.97
2.87
2.04
2.07
f1 (ppm)

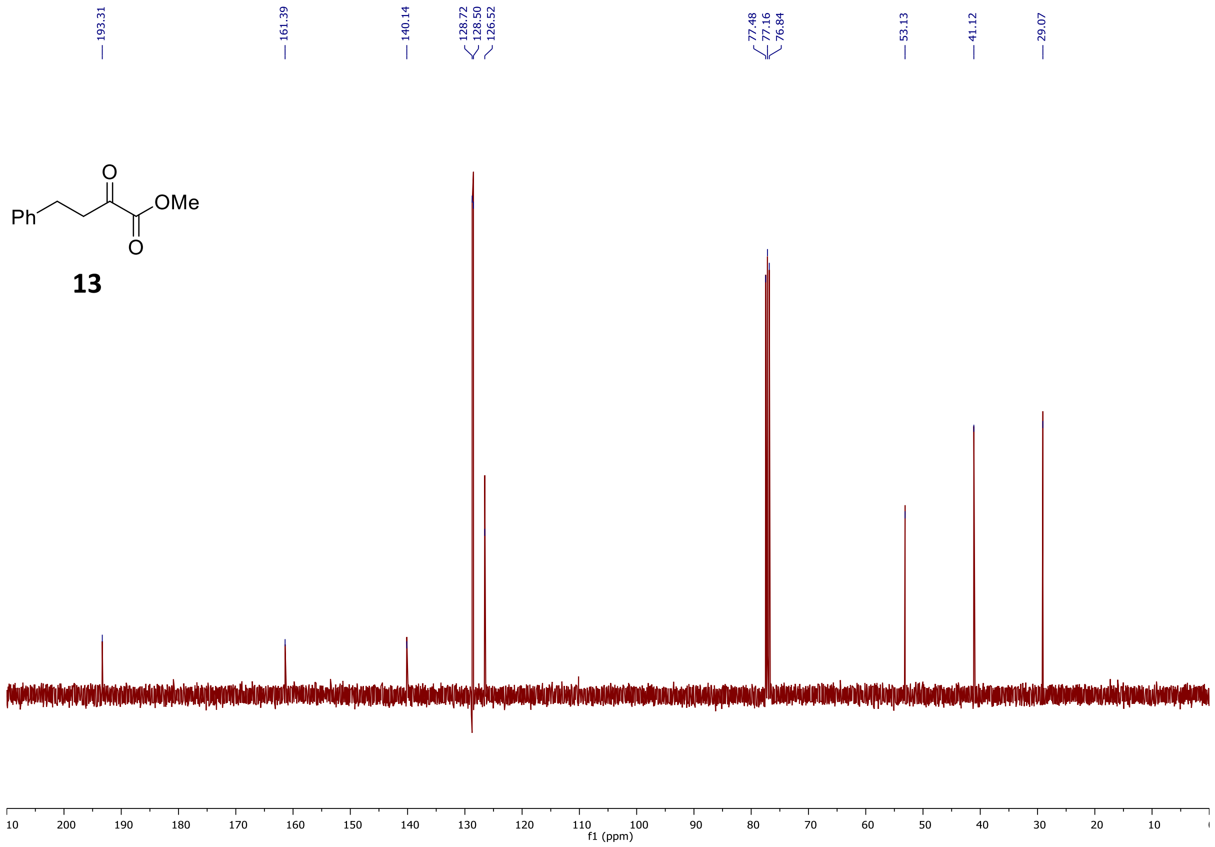
193.31
161.39
140.14
128.72
128.50
126.52
77.48
77.16
76.84
53.13
41.12
29.07
O
OMe
Ph
O
13
f1 (ppm)

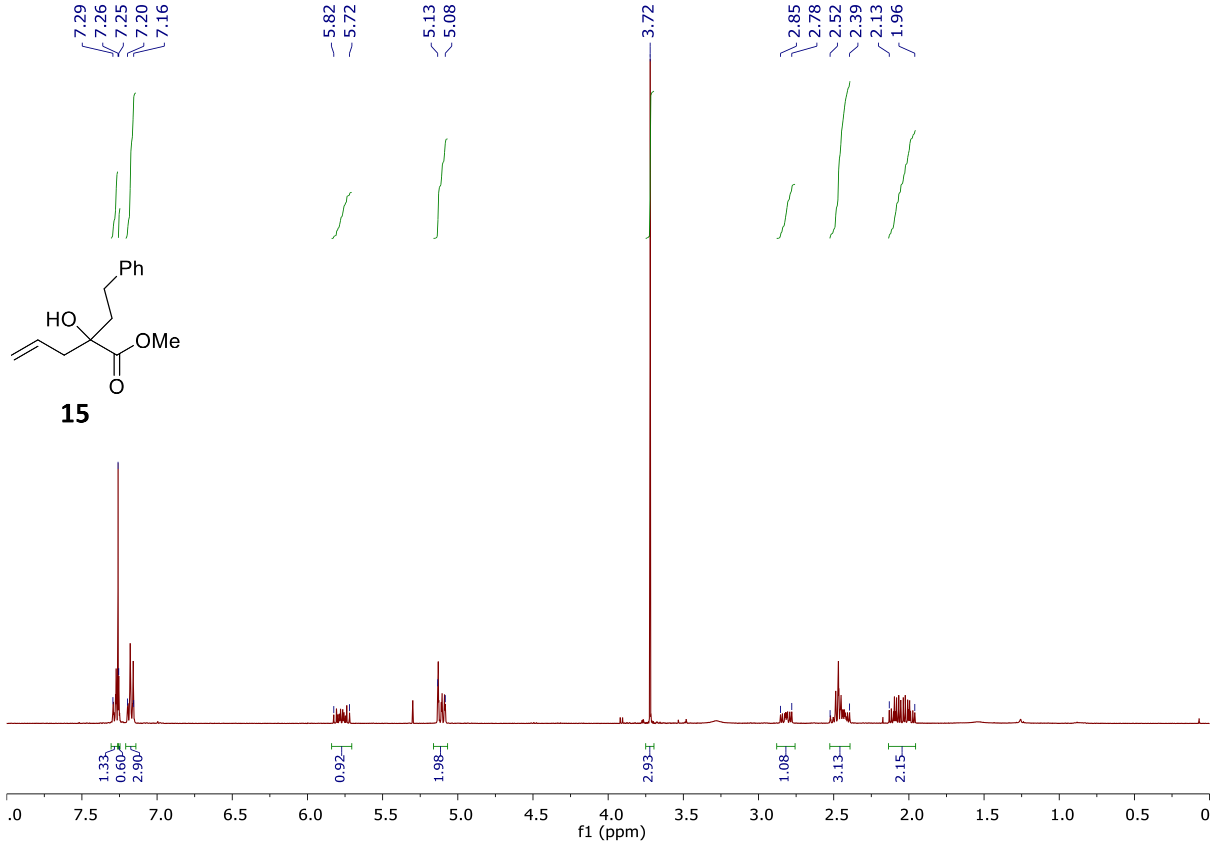
7.29
7.26
7.25
7.20
7.16
5.82
5.72
5.13
5.08
3.72
2.85
2.78
2.52
2.39
2.13
1.96
Ph
HO
OMe
O
15
1.33
0.60
2.90
0.92
1.98
2.93
1.08
3.13
2.15
f1 (ppm)

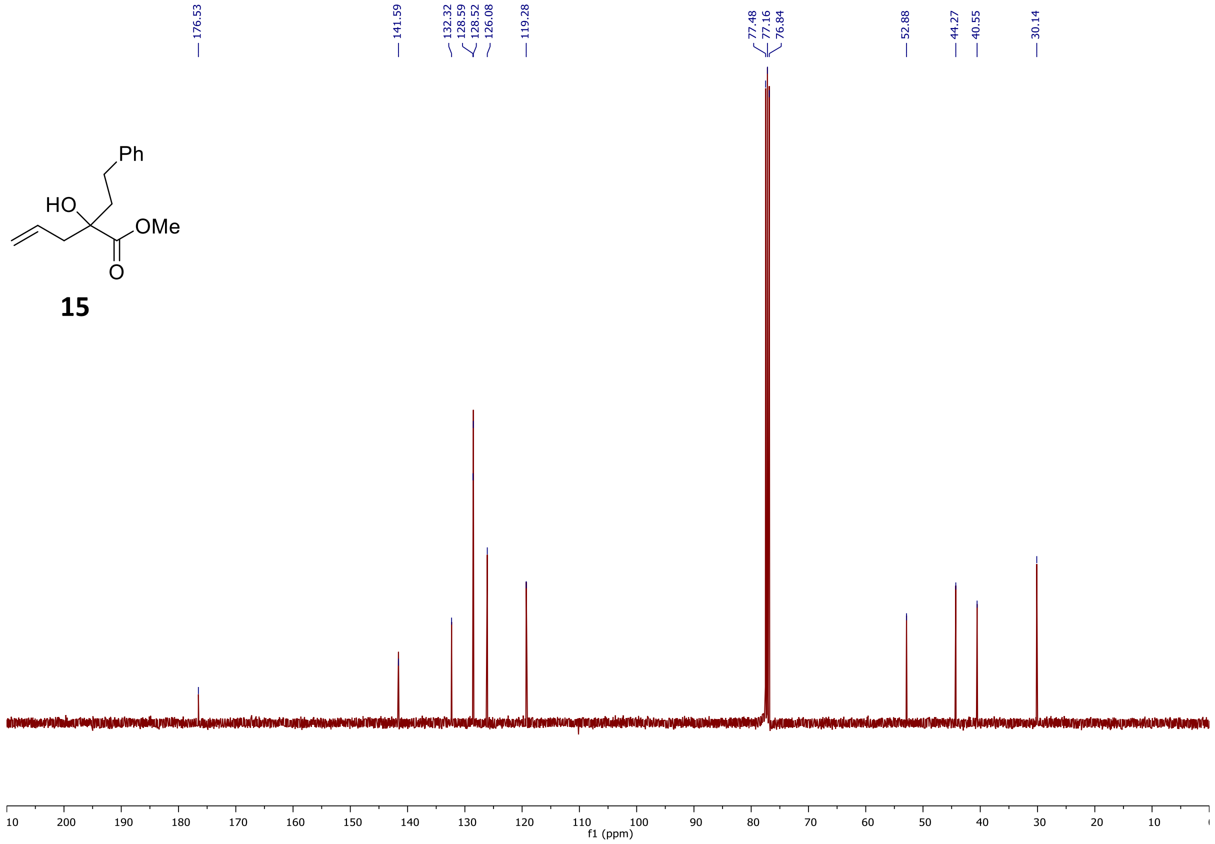
176.53
141.59
132.32
128.59
128.52
126.08
119.28
77.48
77.16
76.84
52.88
44.27
40.55
30.14
Ph
HO
OMe
O
15
f1 (ppm)

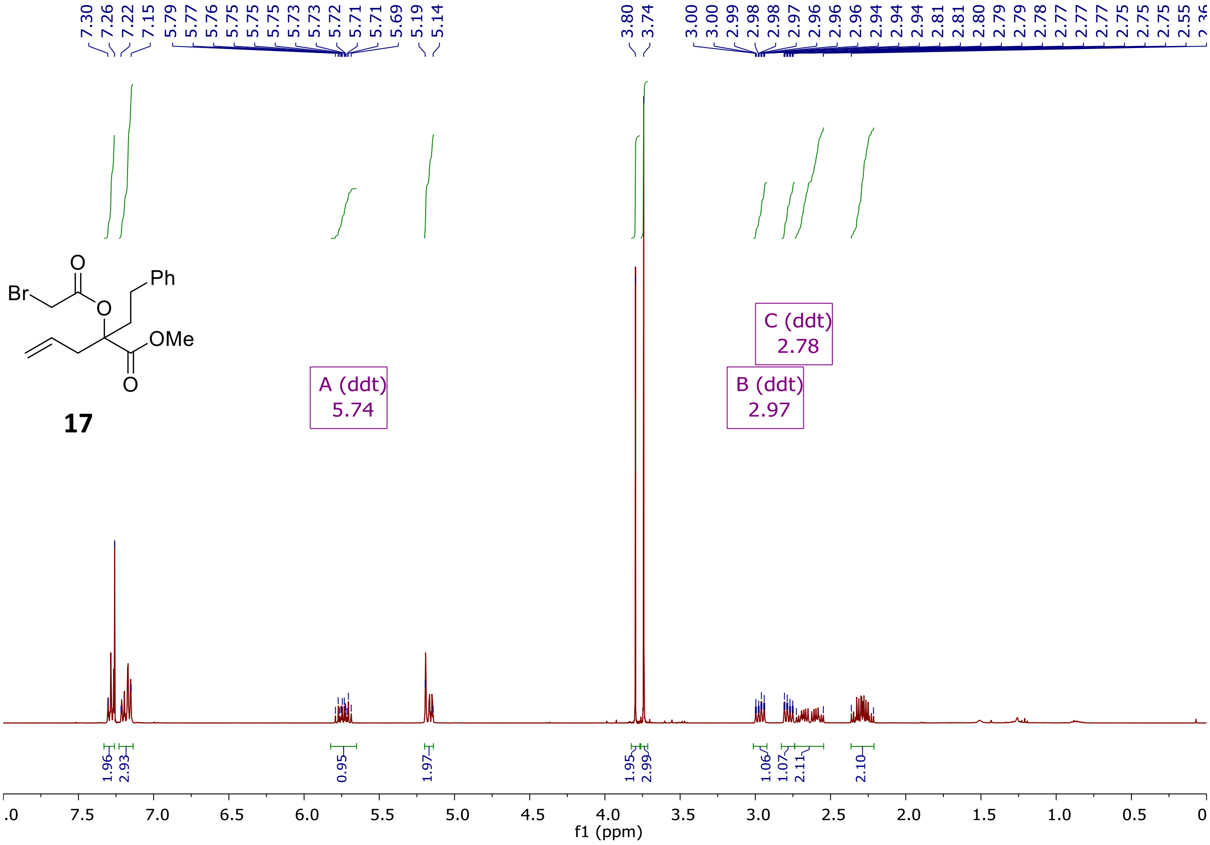

Br
O
Ph
OMe
17
A (ddt)
5.74
B (ddt)
2.97
C (ddt)
2.78
f1 (ppm)

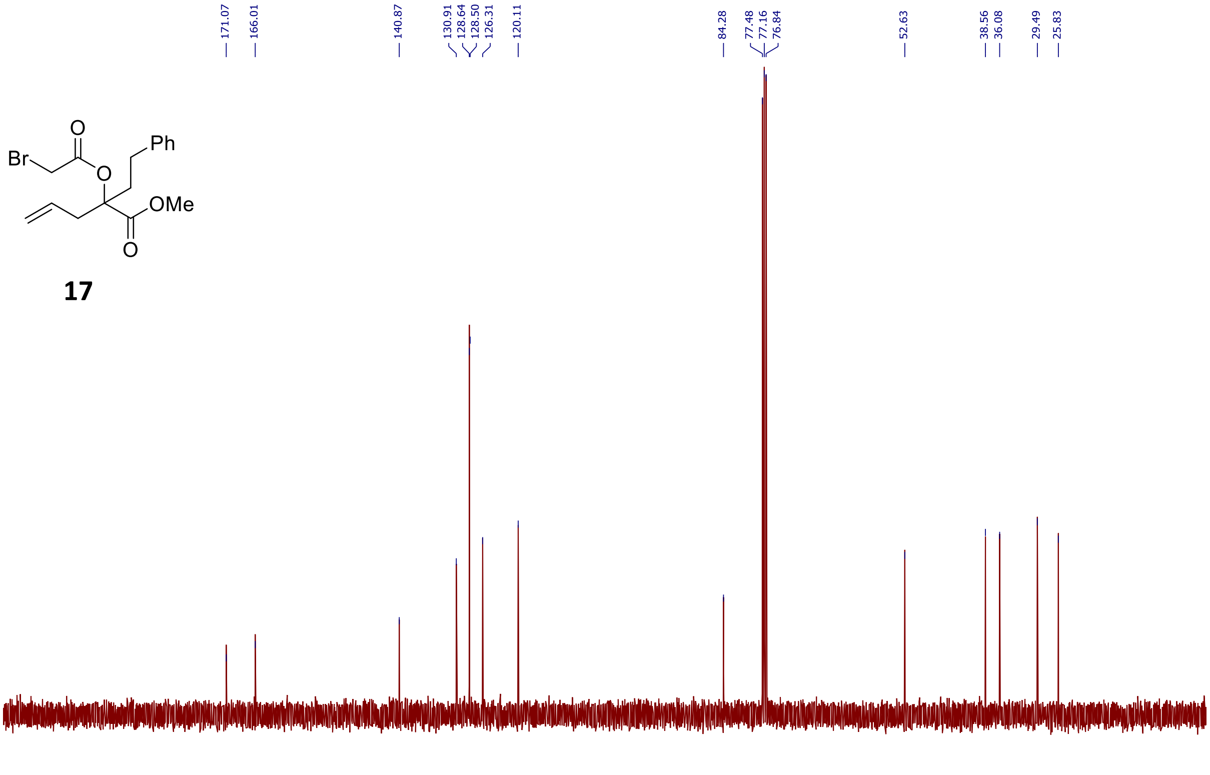

Br
O
Ph
OMe
17
171.07
166.01
140.87
130.91
128.64
128.50
126.31
120.11
84.28
77.48
77.16
76.84
52.63
38.56
36.08
29.49
25.83
f1 (ppm)

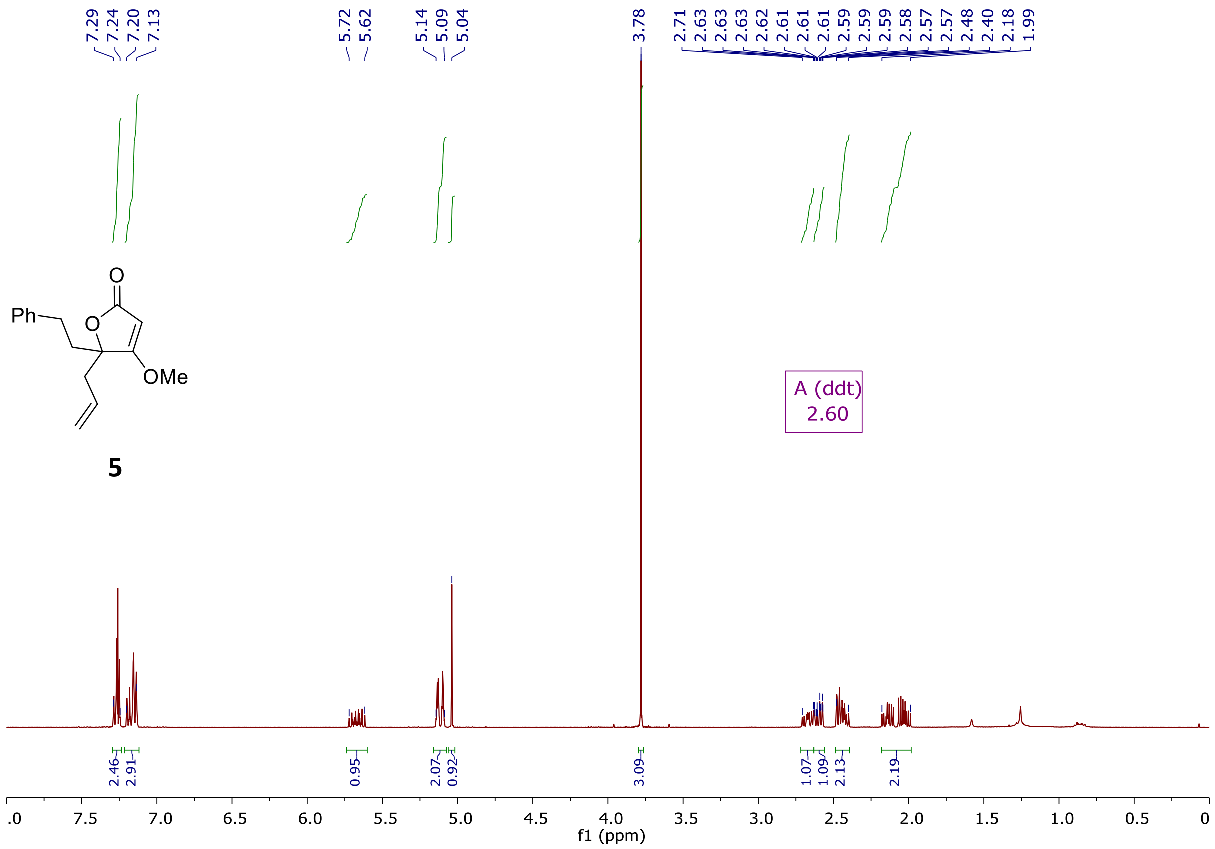
7.29
7.24
7.20
7.13
5.72
5.62
5.14
5.09
5.04
3.78
2.71
2.63
2.63
2.63
2.62
2.61
2.61
2.61
2.61
2.59
2.59
2.59
2.58
2.57
2.57
2.48
2.40
2.18
1.99
Ph
O
O
OMe
5
A (ddt)
2.60
2.46
2.91
0.95
2.07
0.92
3.09
1.07
1.09
2.13
2.19
f1 (ppm)

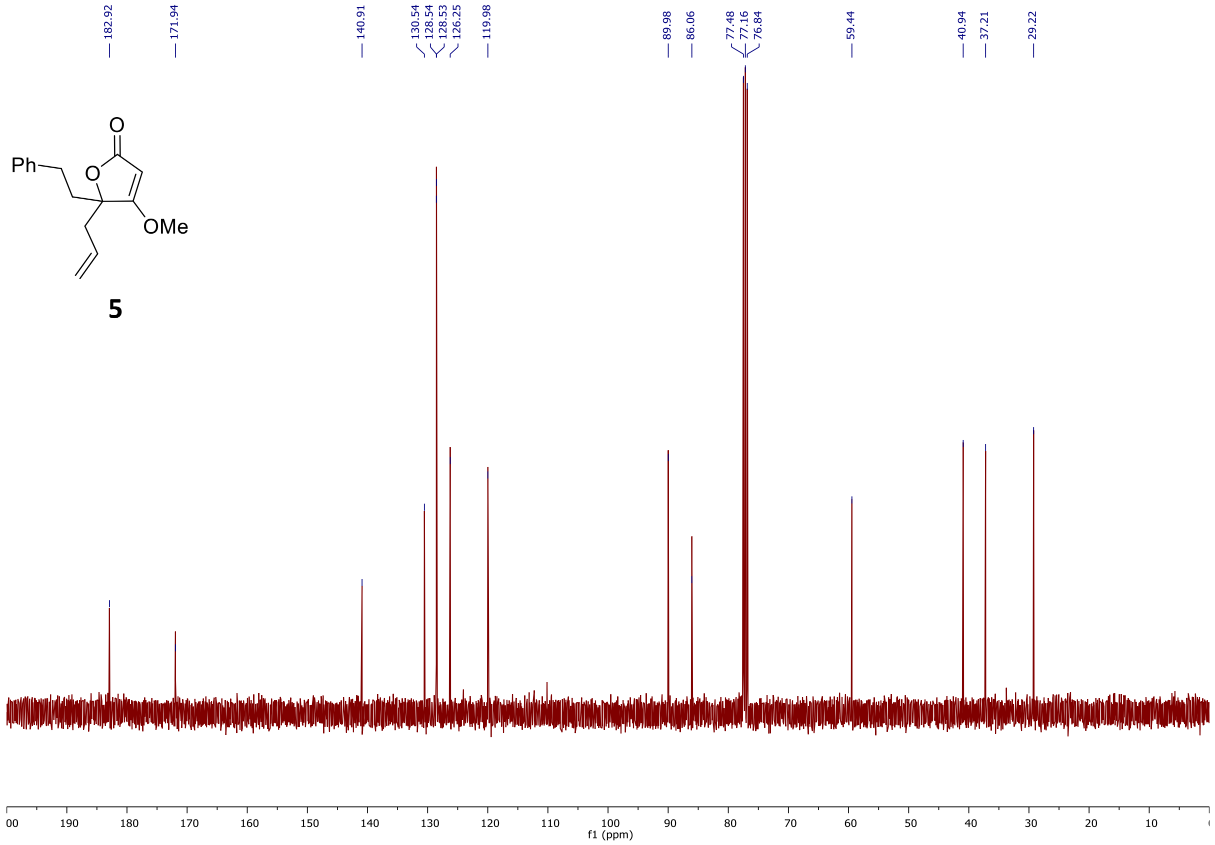
182.92
171.94
140.91
130.54
128.54
128.53
126.25
119.98
89.98
86.06
77.48
77.16
76.84
59.44
40.94
37.21
29.22
Ph
O
O
OMe
5
f1 (ppm)

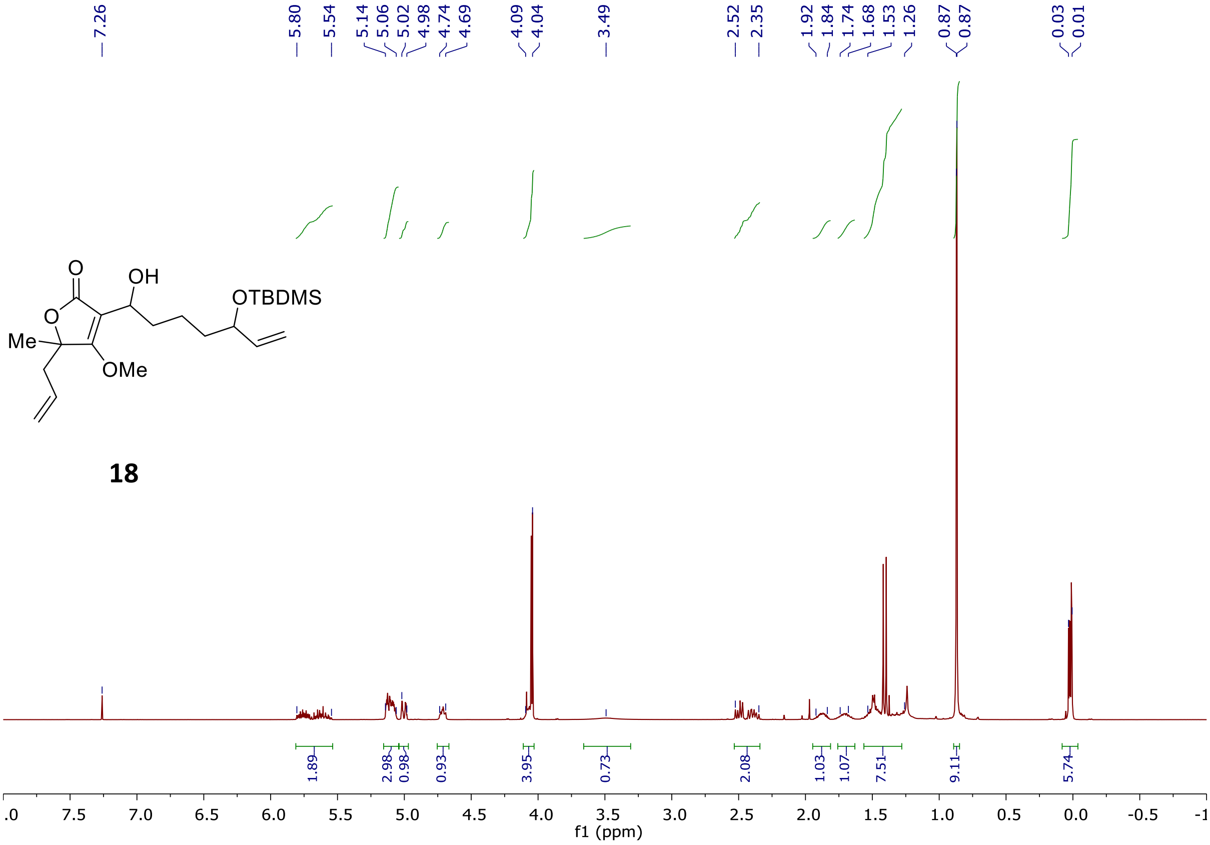
7.26
5.80
5.54
5.14
5.06
5.02
4.98
4.74
4.69
4.09
4.04
3.49
2.52
2.35
1.92
1.84
1.74
1.68
1.53
1.26
0.87
0.87
0.03
0.01
O
OH
OTBDMS
O
Me
OMe
18
1.89
2.98
0.98
0.93
3.95
0.73
2.08
1.03
1.07
7.51
9.11
5.74
f1 (ppm)

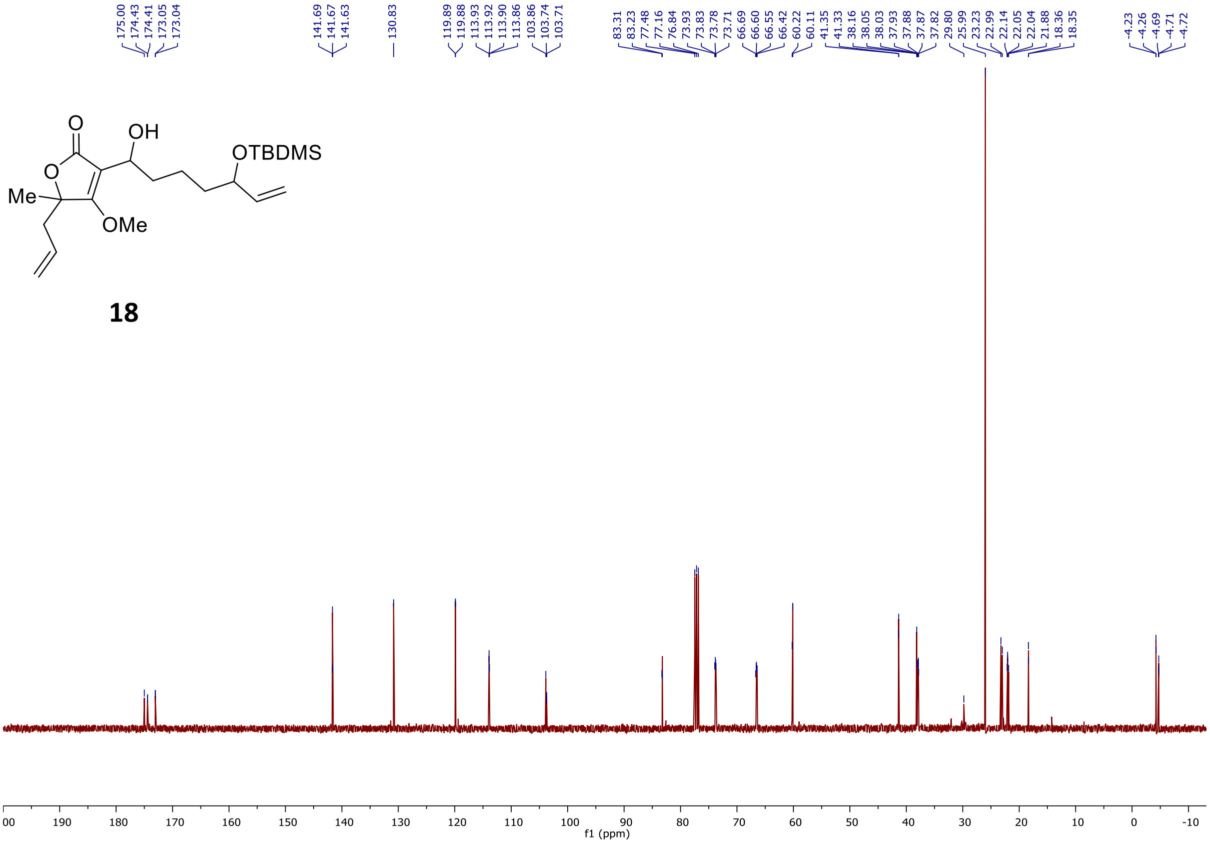
175.00
174.43
174.41
173.05
173.04
141.69
141.67
141.63
130.83
119.89
119.88
113.93
113.92
113.90
113.86
103.86
103.74
103.71
83.31
83.23
77.48
77.16
76.84
73.93
73.83
73.78
73.71
66.69
66.60
66.55
66.42
60.22
60.11
41.35
41.33
38.16
38.05
38.03
37.93
37.88
37.87
37.82
29.80
25.99
23.23
22.99
22.14
22.05
22.04
21.88
18.36
18.35
-4.23
-4.26
-4.69
-4.71
-4.72
O
OH
OTBDMS
O
Me
OMe
18
f1 (ppm)

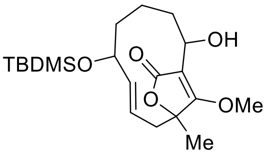
TBDMSO
OH
O
O
OMe
Me

20

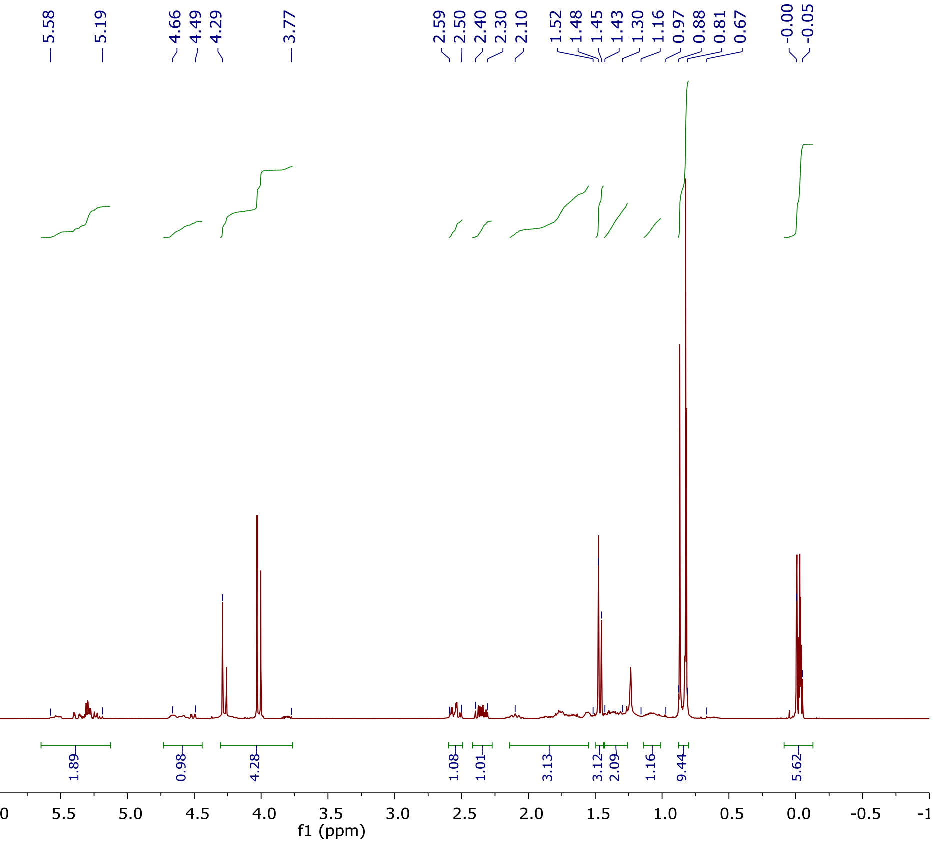
7.26
5.58
5.19
4.66
4.49
4.29
3.77
2.59
2.50
2.40
2.30
2.10
1.52
1.48
1.45
1.43
1.30
1.16
0.97
0.88
0.81
0.67
-0.00
-0.05
1.89
0.98
4.28
1.08
1.01
3.13
3.12
2.09
1.16
9.44
5.62
f1 (ppm)

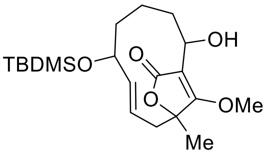
TBDMSO
OH
O
O
OMe
Me

20

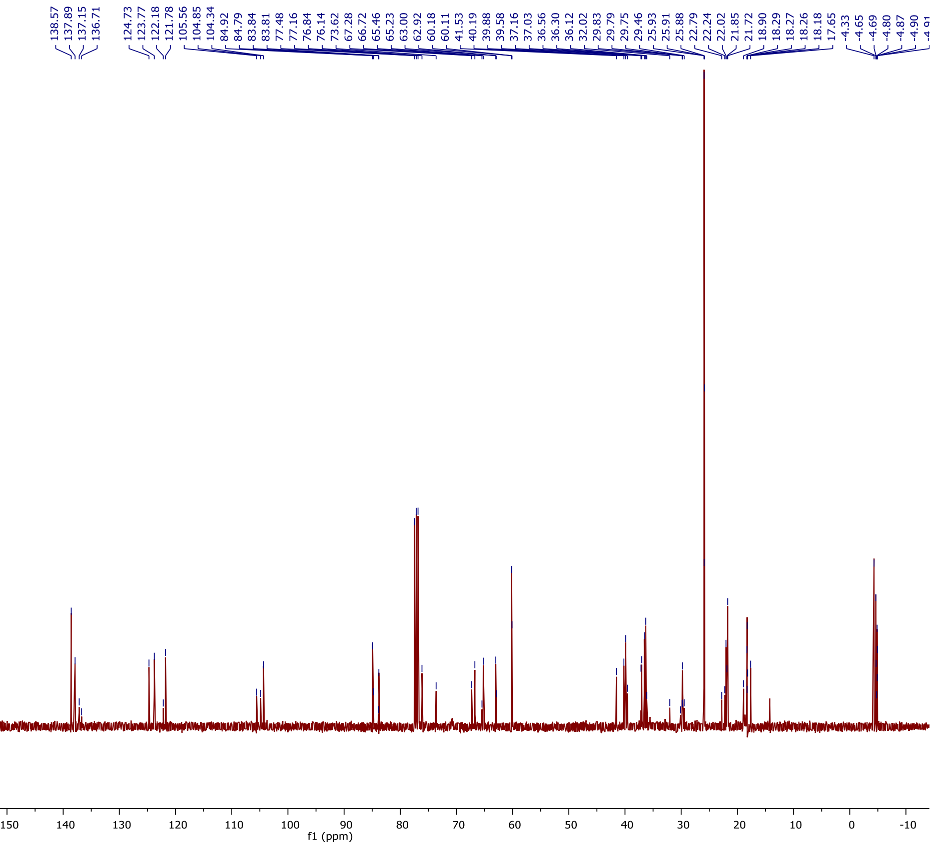
178.68
178.44
174.71
174.12
173.94
173.88
173.62
138.57
137.89
137.15
136.71
124.73
123.77
122.18
121.78
105.56
104.85
104.34
84.92
84.79
83.84
83.81
77.48
77.16
76.84
76.14
73.62
67.28
66.72
65.46
65.23
63.00
62.92
60.18
60.11
41.53
40.19
39.88
39.58
37.16
37.03
36.56
36.30
36.12
32.02
29.83
29.79
29.75
29.46
25.93
25.91
25.88
22.79
22.24
22.02
21.85
21.72
18.90
18.29
18.27
18.26
18.18
17.65
-4.33
-4.65
-4.69
-4.80
-4.87
-4.90
f1 (ppm)

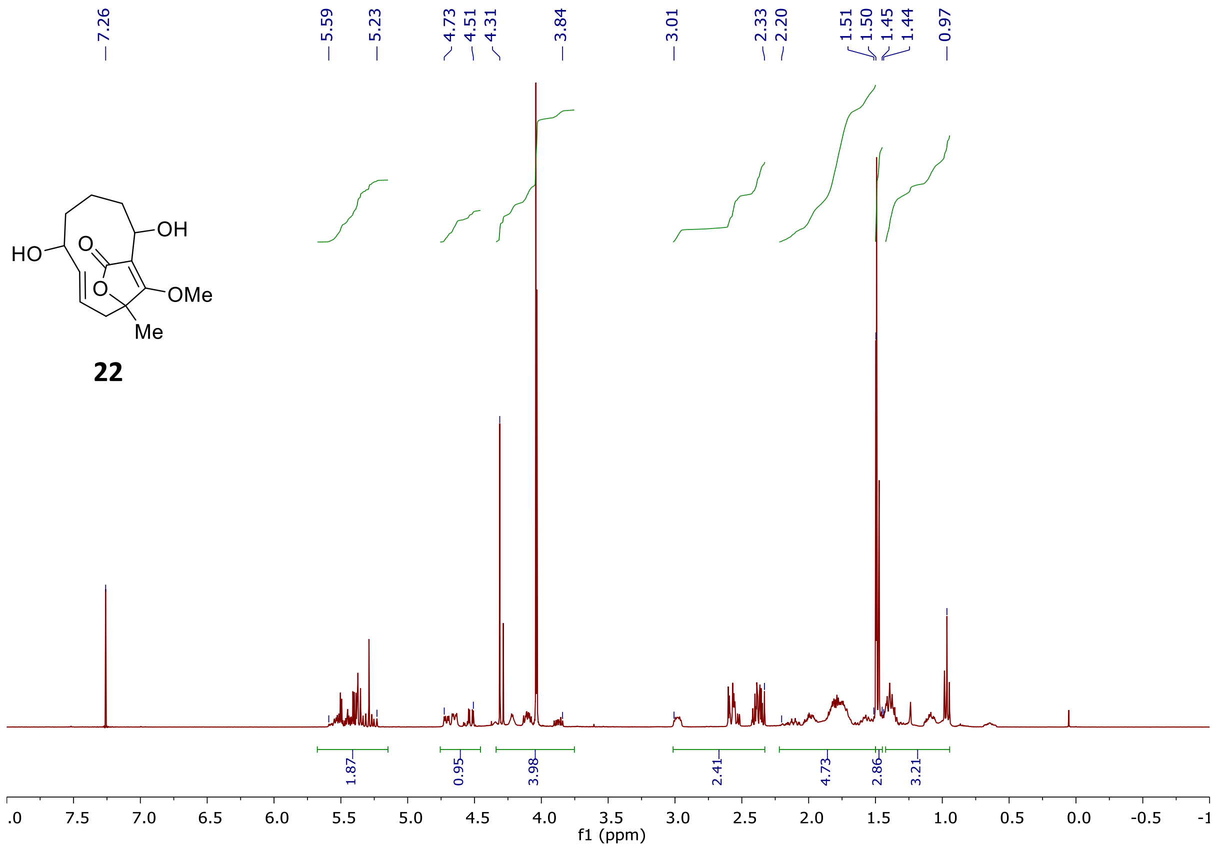

7.26
5.59
5.23
4.73
4.51
4.31
3.84
3.01
2.33
2.20
1.51
1.50
1.45
1.44
0.97
HO
OH
O
O
OMe
Me
22
1.87
0.95
3.98
2.41
4.73
2.86
3.21
f1 (ppm)

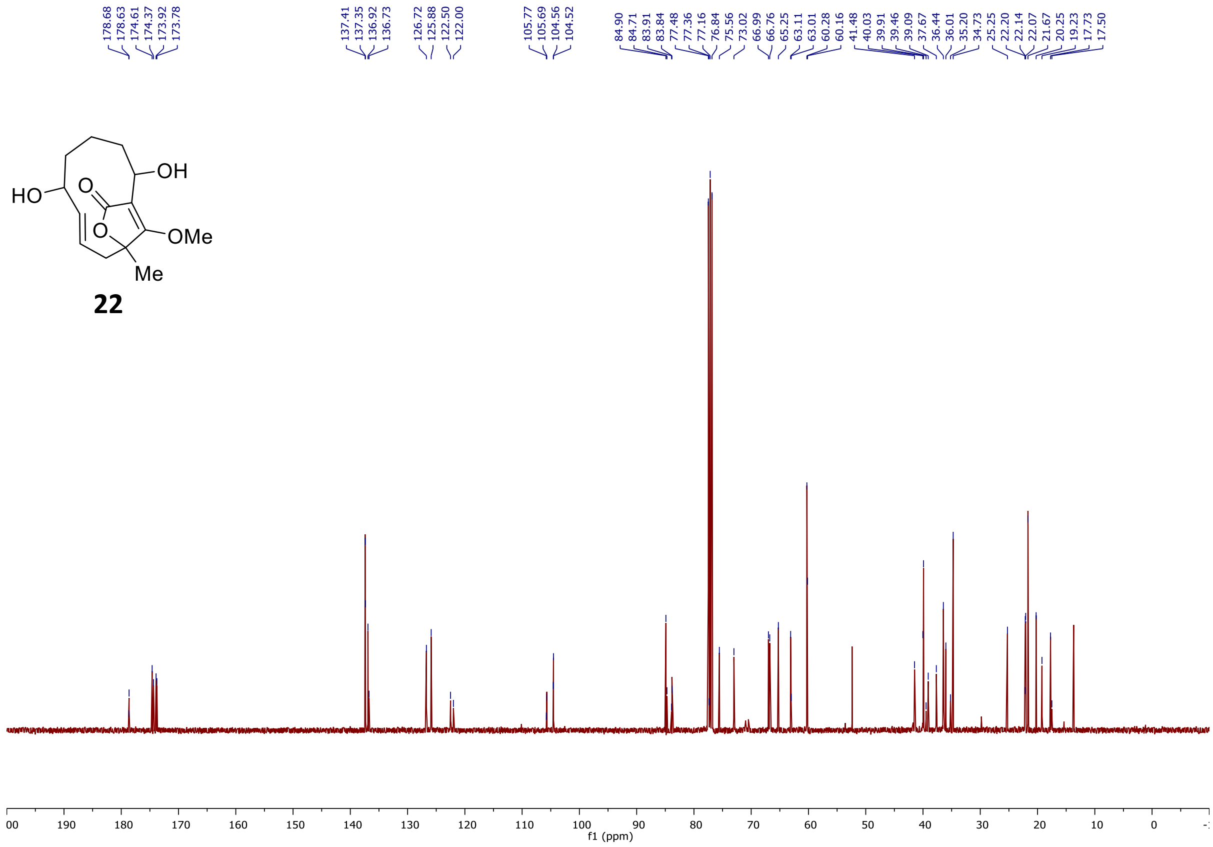

178.68
178.63
174.61
174.37
173.92
173.78
137.41
137.35
136.92
136.73
126.72
125.88
122.50
122.00
105.77
105.69
104.56
104.52
84.90
84.71
83.91
83.84
77.48
77.36
77.16
76.84
75.56
73.02
66.99
66.76
65.25
63.11
63.01
60.28
60.16
41.48
40.03
39.91
39.46
39.09
37.67
36.44
36.01
35.20
34.73
25.25
22.20
22.14
22.07
21.67
20.25
19.23
17.73
17.50
HO
OH
O
O
OMe
Me
22
f1 (ppm)

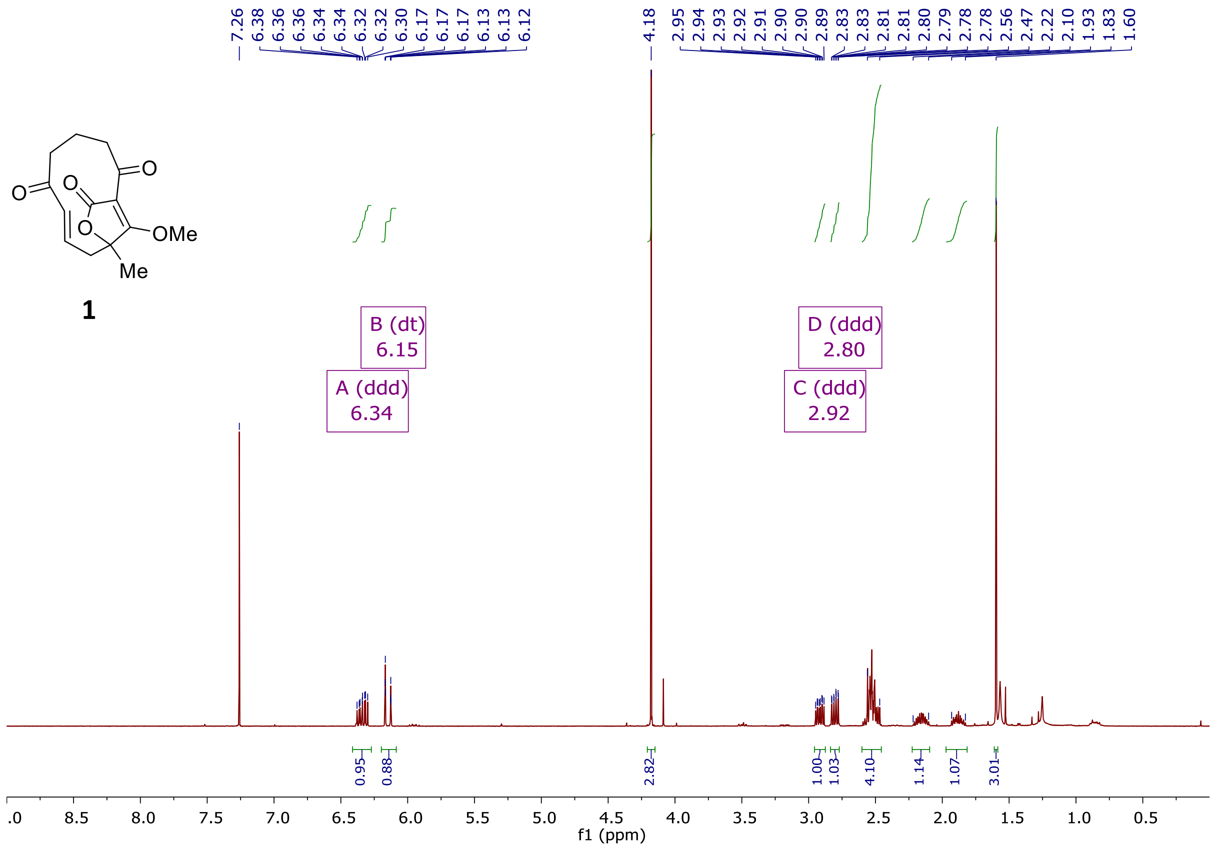

7.26
6.38 6.36 6.36 6.34 6.34 6.32 6.32 6.30 6.17 6.17 6.17 6.13 6.13 6.12
4.18
2.95 2.94 2.93 2.92 2.91 2.90 2.90 2.89 2.83 2.83 2.81 2.81 2.80 2.79 2.78 2.78 2.56 2.47 2.22 2.10 1.93 1.83 1.60
OMe
Me
1
B (dt) 6.15
A (ddd) 6.34
D (ddd) 2.80
C (ddd) 2.92
0.95 0.88 2.82 1.00 1.03 4.10 1.14 1.07 3.01
f1 (ppm)

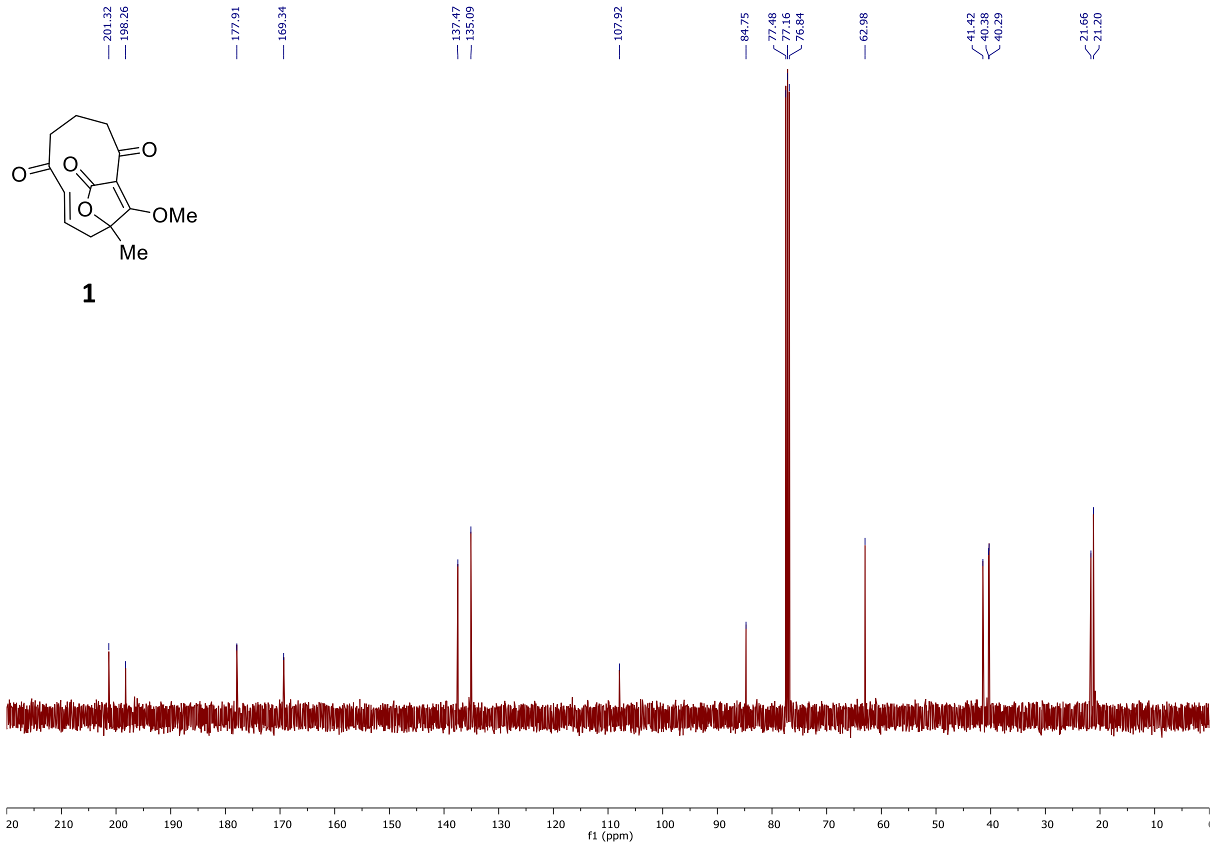

201.32 198.26 177.91 169.34 137.47 135.09 107.92 84.75 77.48 77.16 76.84 62.98 41.42 40.38 40.29 21.66 21.20
OMe
Me
1
f1 (ppm)

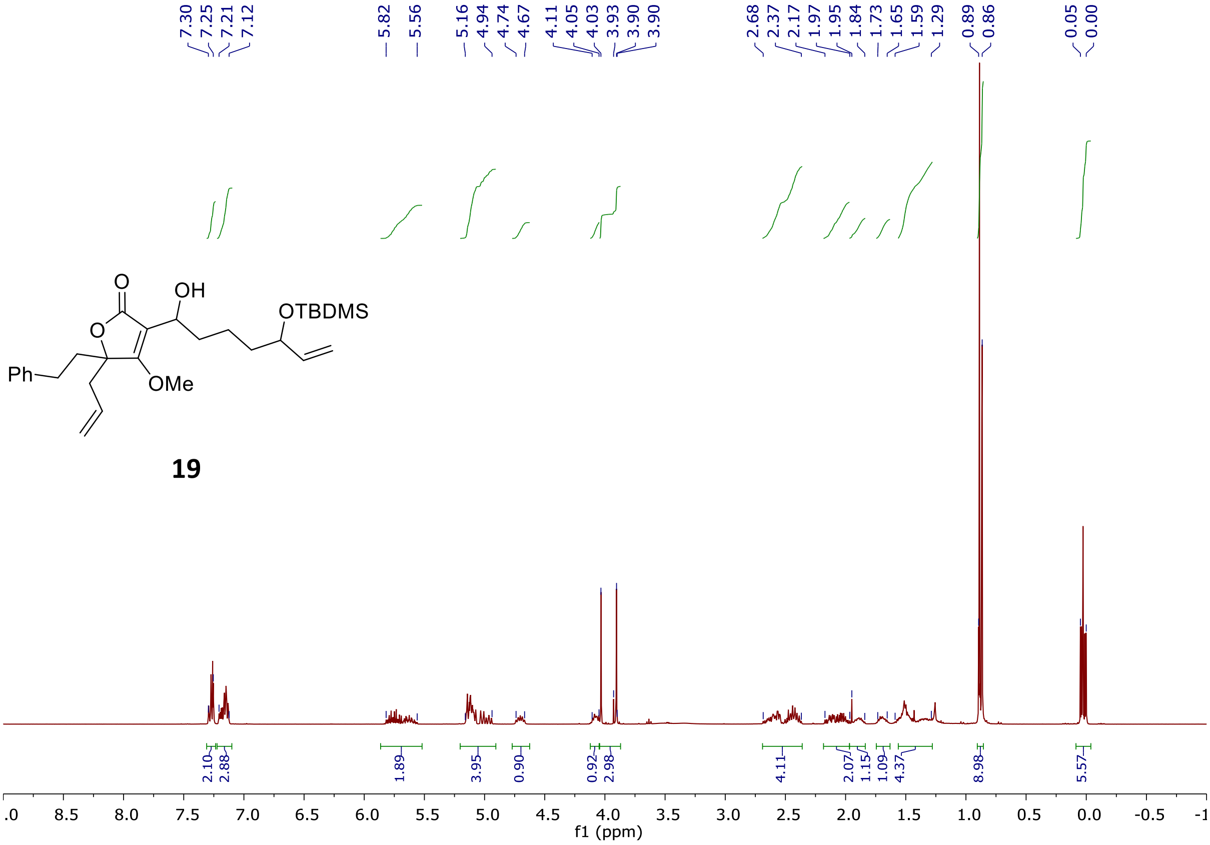
7.30
7.25
7.21
7.12
5.82
5.56
5.16
4.94
4.74
4.67
4.11
4.05
4.03
3.93
3.90
3.90
2.68
2.37
2.17
1.97
1.95
1.84
1.73
1.65
1.59
1.29
0.89
0.86
0.05
0.00
O
OH
OTBDMS
O
Ph
OMe
19
2.10
2.88
1.89
3.95
0.90
0.92
2.98
4.11
2.07
1.15
1.09
4.37
8.98
5.57
f1 (ppm)

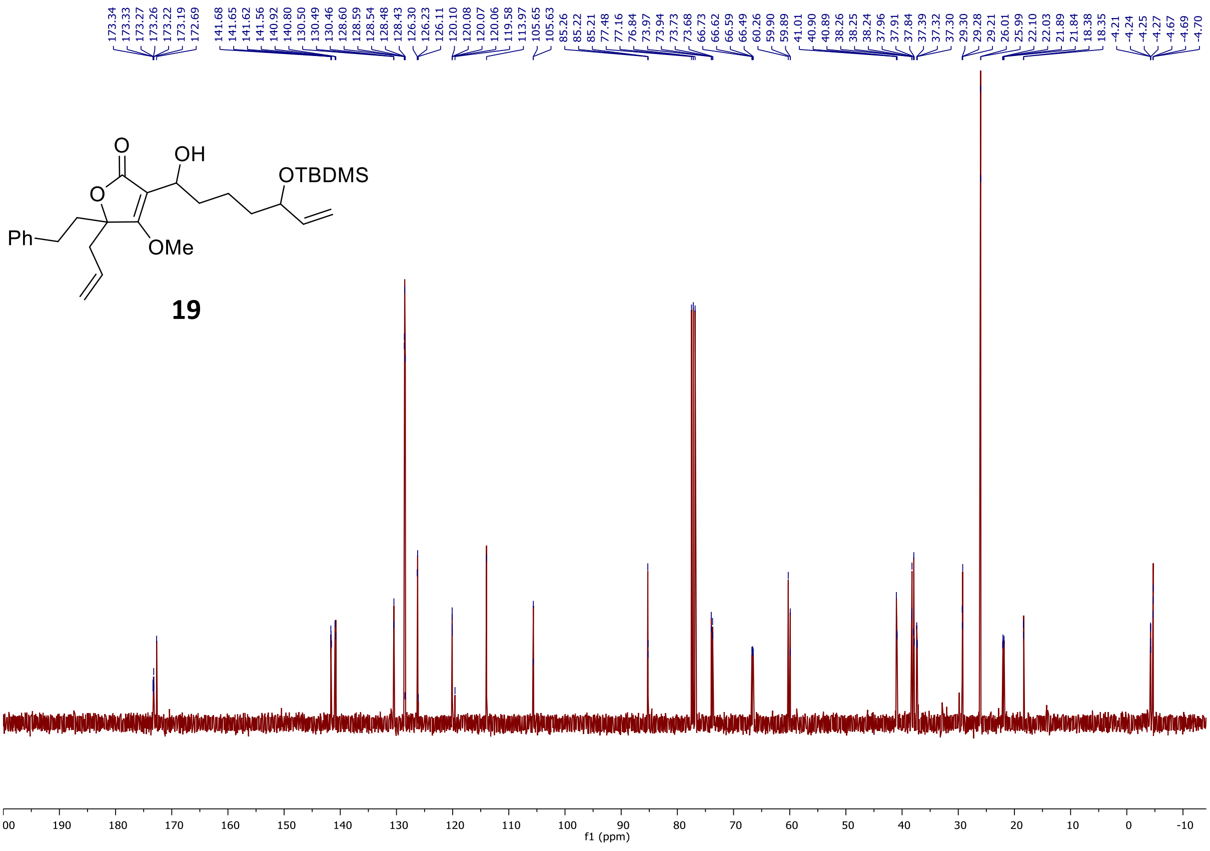
173.34
173.33
173.27
173.26
173.22
173.19
172.69
141.68
141.65
141.62
141.56
140.92
140.80
130.50
130.49
130.46
128.60
128.59
128.54
128.48
128.43
126.30
126.23
126.11
120.10
120.08
120.07
120.06
119.58
113.97
105.65
105.63
85.26
85.22
85.21
77.48
77.16
76.84
73.97
73.94
73.73
73.68
66.73
66.62
66.59
66.49
60.26
59.90
59.89
41.01
40.90
40.89
38.26
38.25
38.24
37.96
37.91
37.84
37.39
37.32
37.30
29.30
29.28
29.21
26.01
25.99
22.10
22.03
21.89
21.84
18.38
18.35
-4.21
-4.24
-4.25
-4.27
-4.67
-4.69
-4.70
O
OH
OTBDMS
O
Ph
OMe
19
f1 (ppm)

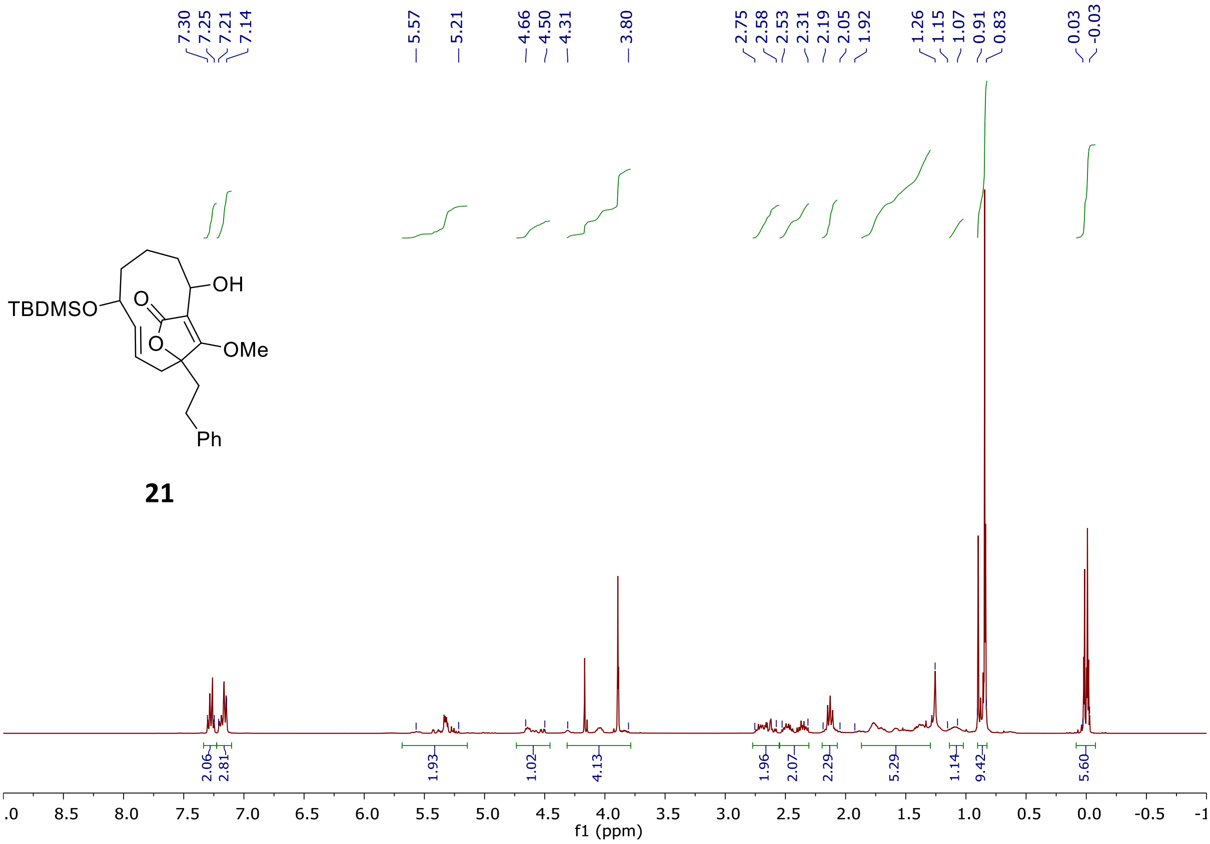
7.30
7.25
7.21
7.14
5.57
5.21
4.66
4.50
4.31
3.80
2.75
2.58
2.53
2.31
2.19
2.05
1.92
1.26
1.15
1.07
0.91
0.83
0.03
-0.03
TBDMSO
OH
O
O
OMe
Ph
21
2.06
2.81
1.93
1.02
4.13
1.96
2.07
2.29
5.29
1.14
9.42
5.60
f1 (ppm)

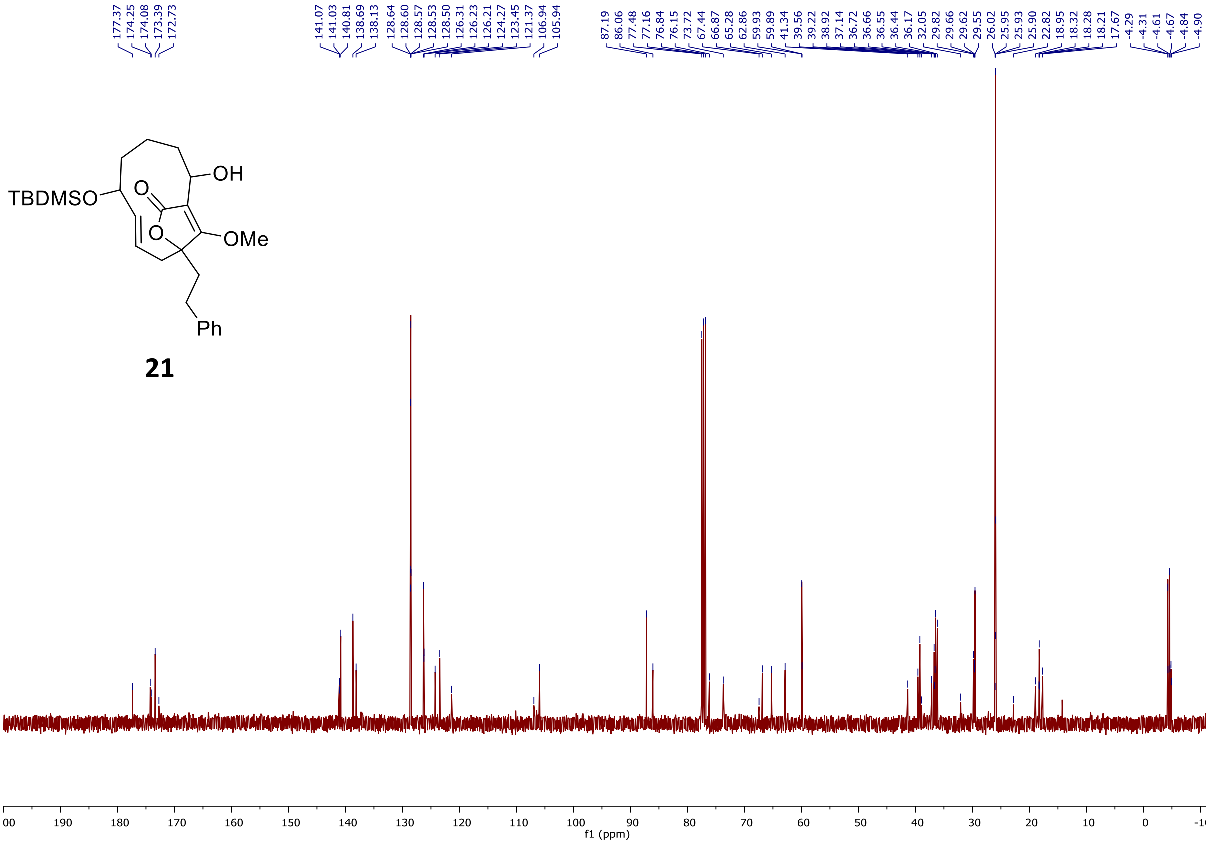
177.37
174.25
174.08
173.39
172.73
141.07
141.03
140.81
138.69
138.13
128.64
128.60
128.57
128.53
128.50
126.31
126.23
126.21
124.27
123.45
121.37
106.94
105.94
87.19
86.06
77.48
77.16
76.84
76.15
73.72
67.44
66.87
65.28
62.86
59.93
59.89
41.34
39.56
39.22
38.92
37.14
36.72
36.66
36.55
36.44
36.17
32.05
29.82
29.66
29.62
29.55
26.02
25.95
25.93
25.90
22.82
18.95
18.32
18.28
18.21
17.67
-4.29
-4.31
-4.61
-4.67
-4.84
-4.90
TBDMSO
OH
O
O
OMe
Ph
21
f1 (ppm)

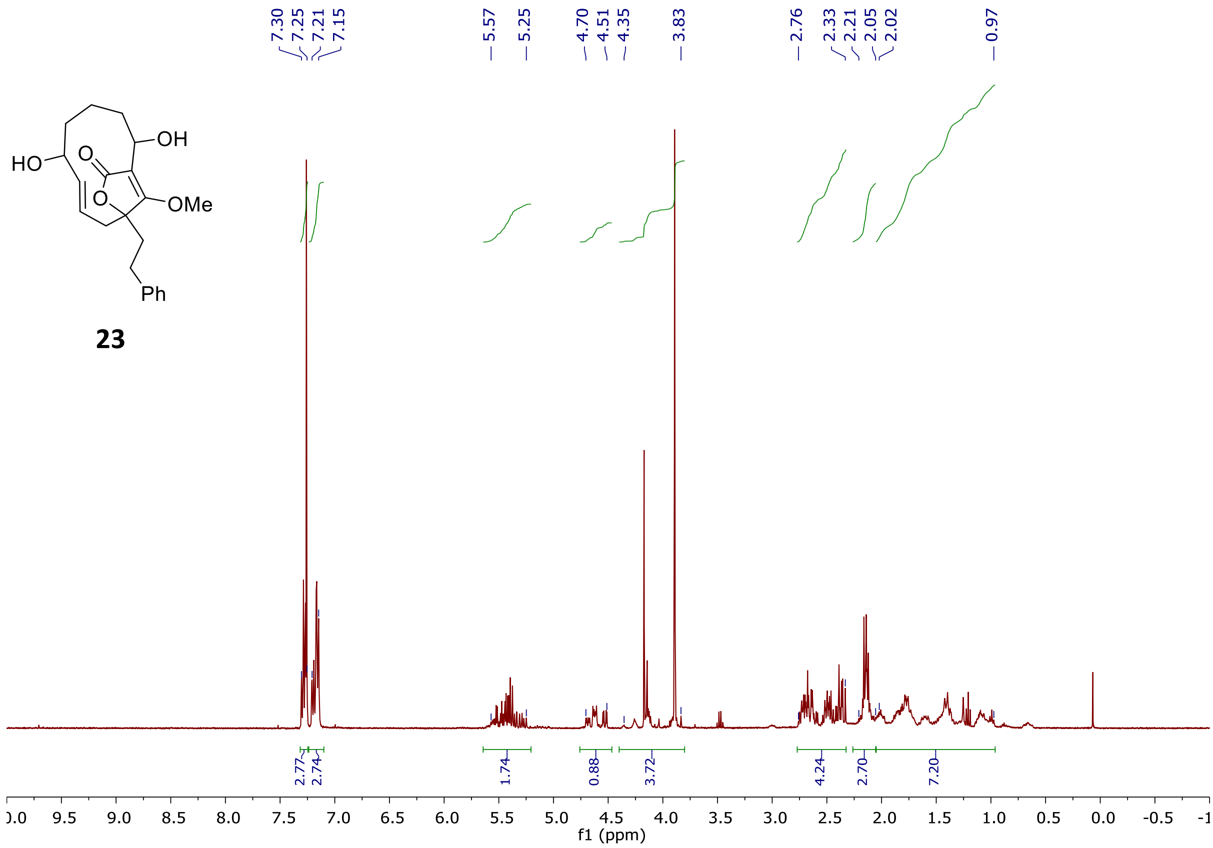
7.30
7.25
7.21
7.15
5.57
5.25
4.70
4.51
4.35
3.83
2.76
2.33
2.21
2.05
2.02
0.97
HO
OH
O
O
OMe
Ph
23
2.77
2.74
1.74
0.88
3.72
4.24
2.70
7.20
f1 (ppm)

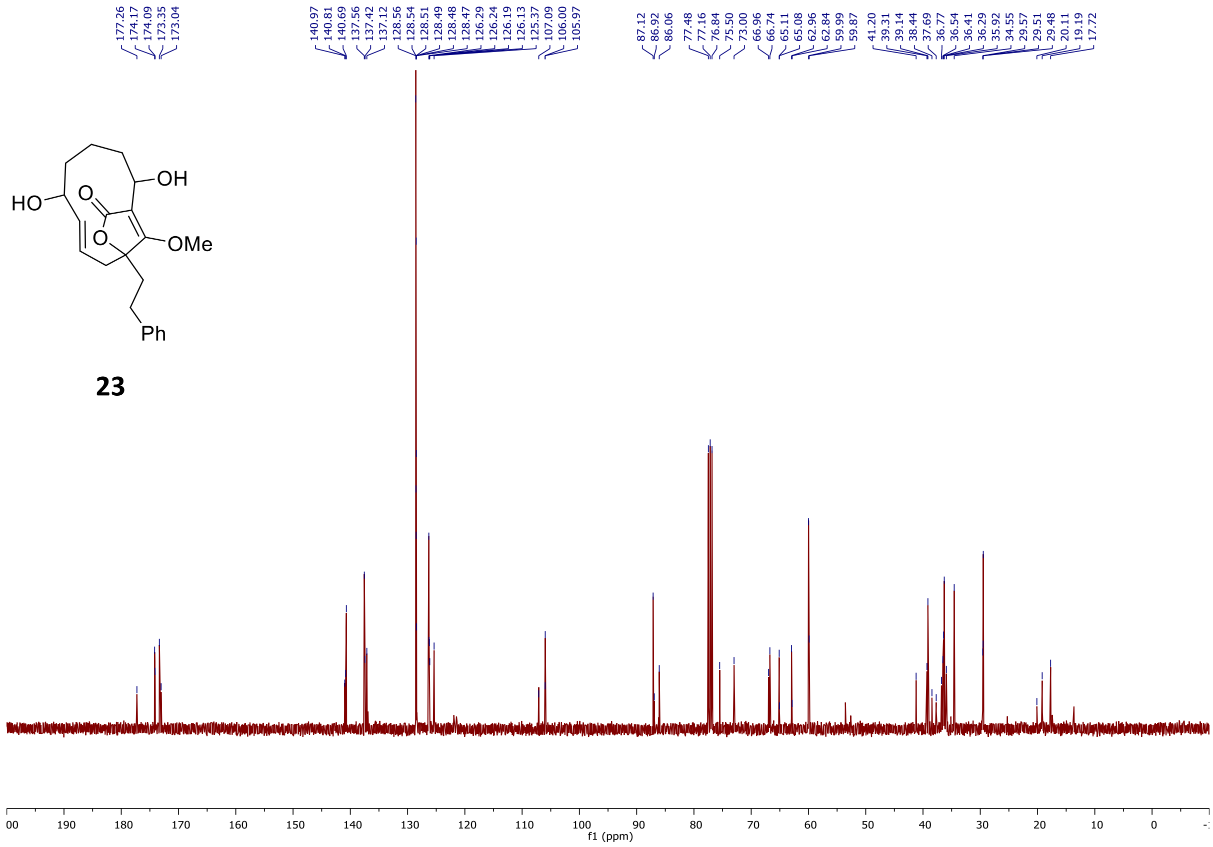
177.26
174.17
174.09
173.35
173.04
140.97
140.81
140.69
137.56
137.42
137.12
128.56
128.54
128.51
128.49
128.48
128.47
126.29
126.24
126.19
126.13
125.37
107.09
106.00
105.97
87.12
86.92
86.06
77.48
77.16
76.84
75.50
73.00
66.96
66.74
65.11
65.08
62.96
62.84
59.99
59.87
41.20
39.31
39.14
38.44
37.69
36.77
36.54
36.41
36.29
35.92
34.55
29.57
29.51
29.48
20.11
19.19
17.72
HO
OH
O
O
OMe
Ph
23
f1 (ppm)

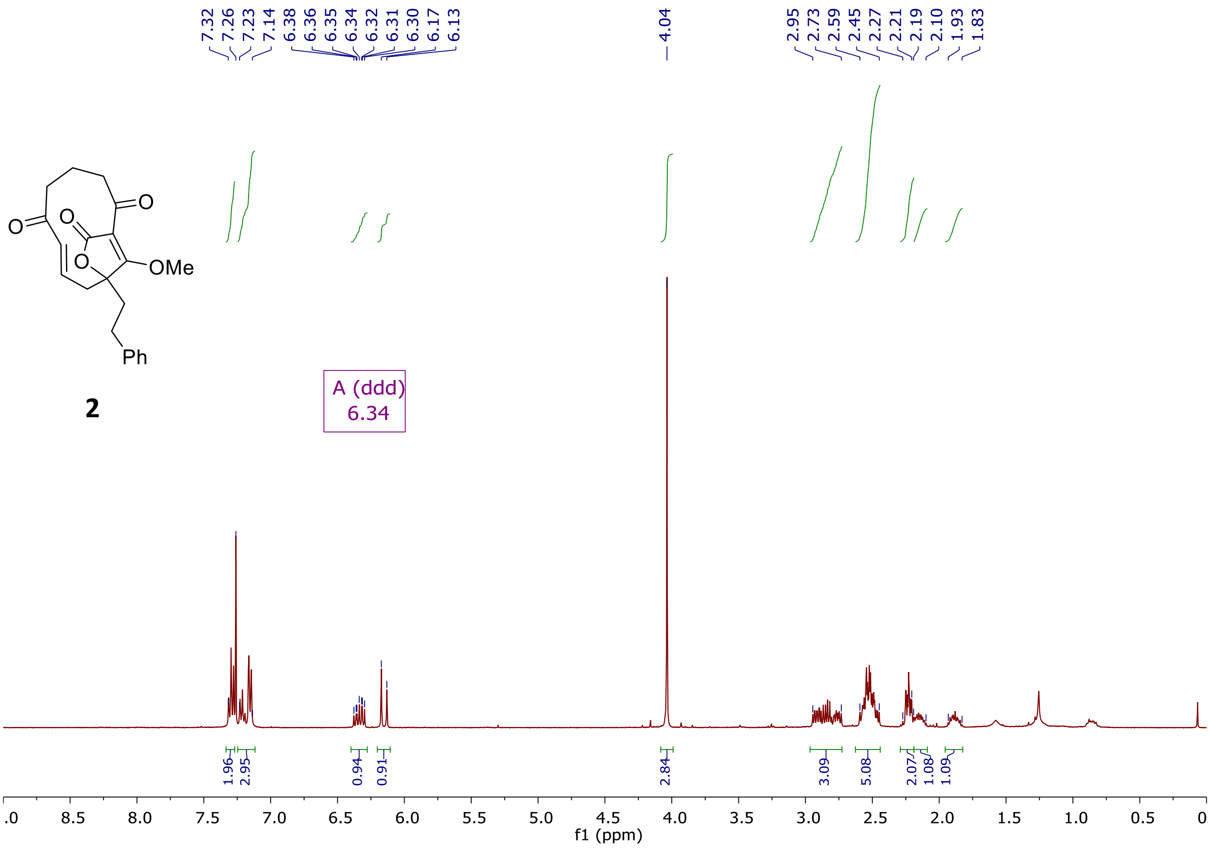
7.32
7.26
7.23
7.14
6.38
6.36
6.35
6.34
6.32
6.31
6.30
6.17
6.13
4.04
2.95
2.73
2.59
2.45
2.27
2.21
2.19
2.10
1.93
1.83
O
O
O
O
OMe
Ph
2
A (ddd)
6.34
1.96
2.95
0.94
0.91
2.84
3.09
5.08
2.07
1.08
1.09
f1 (ppm)

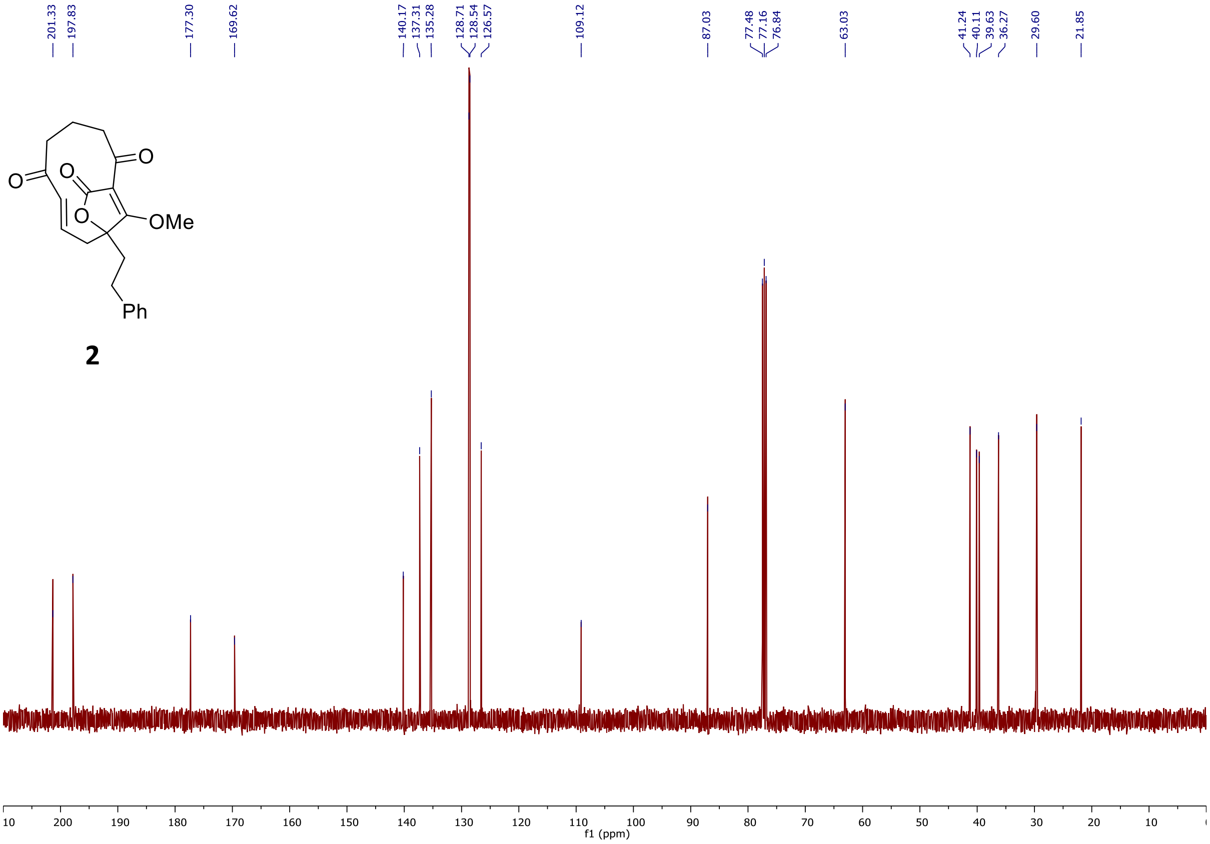
201.33
197.83
177.30
169.62
140.17
137.31
135.28
128.71
128.54
126.57
109.12
87.03
77.48
77.16
76.84
63.03
41.24
40.11
39.63
36.27
29.60
21.85
O
O
O
O
OMe
Ph
2
f1 (ppm)
